# Network analysis in depressed adolescents with suicidal ideation: the role of depression, anxiety, and childhood abuse

**DOI:** 10.3389/fpsyt.2025.1645303

**Published:** 2025-08-08

**Authors:** Ziyang Huang, Liya A, Kewen Yan, Hailiang Ran, Yusan Che, Runxu Yang, Linling Jiang, Rui Xiao, Rushuang Zeng, Tong Li, Yiling Xie, Yuanyuan Xiao, Jin Lu

**Affiliations:** ^1^ Psychiatric Department, First Affiliated Hospital of Kunming Medical University, Kunming, China; ^2^ Mental Health Institute of Yunnan, First Affiliated Hospital of Kunming Medical University, Kunming, China; ^3^ Yunnan Clinical Research Center for Mental Health, Kunming, China; ^4^ Taylor’s University, Subang Jaya, Selangor, Malaysia; ^5^ School of Public Health, Kunming Medical University, Kunming, China; ^6^ Department of Pediatrics, The First People’s Hospital of Yunnan Province, Kunming, China; ^7^ Department of Pediatrics, The Affiliated Hospital of Kunming University of Science and Technology, Kunming, China

**Keywords:** depressed adolescents, network analysis, suicidal ideation, childhood abuse, depression and anxiety symptoms

## Abstract

**Introduction:**

Adolescent depression is a global public health issue strongly associated with suicidal ideation and childhood abuse. Although family systems and ecological theories highlight the multilevel influences of family environment on mental health, most studies focus on overall symptom scores rather than examining how specific forms of abuse relate to distinct symptoms. Employing symptom network analysis, this study investigates the interactions among depressive symptoms, anxiety, and childhood abuse in adolescents diagnosed with major depressive disorder (MDD) and suicidal ideation.

**Methods:**

We analyzed data from 733 Chinese adolescents diagnosed with MDD (mean age = 14.81 years). Symptom networks were constructed via LASSO-regularized models using the Patient Health Questionnaire-9 (PHQ-9), Generalized Anxiety Disorder-7 (GAD-7), and the Childhood Trauma Questionnaire-Short Form (CTQ-SF). Centrality (strength and bridge strength) and stability analysis identified core symptoms and bridging pathways.

**Results:**

Depressive and anxiety symptoms showed strong comorbidity, with “Uncontrollable worry” (GAD2) and “Fatigue” (PHQ4) as central nodes. Key bridge symptoms included “Motor” (PHQ8), “Death” (PHQ9), “Restless” (GAD5), and “Emotional abuse” (EA). Childhood abuse exhibited intra-group correlations (emotional-physical abuse), and emotional abuse was directly linked to death-related thoughts. The network demonstrated strong stability.

**Conclusions:**

Emotional abuse and bridge symptoms (e.g., fatigue and uncontrolled worry) are critical intervention targets for suicide−prevention interventions. A multimodal approach integrating cognitive-behavioral therapy for core symptom management, family-based interventions to address attachment disruptions, and policy initiatives to reduce childhood abuse is recommended.

## 1 Introduction

Major Depressive Disorder (MDD) is a prevalent mental health condition, affecting an estimated 280 million individuals globally (1). However, treatment rates remain notably low (2). The onset of MDD during childhood and adolescence significantly impacts physical development, academic performance, and interpersonal relationships (3). According to recent survey data, approximately 14.8% of adolescents in China are at risk of depression (4). Notably, the prevalence of depressive symptoms in this population has markedly increased compared to pre-COVID-19 levels (5). In addition, suicidal ideation is highly prevalent among adolescents with MDD (6) and may predict increased risk of suicidal behavior or progression to severe mental illness in adulthood (7). Globally, suicide ranks as the fourth leading cause of death among individuals aged 15 to 29 years (8).

Suicidal ideation, defined as thoughts of ending one’s life without immediate action, is closely associated with the severity of depressive symptoms (9). In China, the prevalence of suicidal ideation among children and adolescents is reported to be 15.4% (10), with 60–70 % of adolescents experiencing depression also exhibiting suicidal ideation (11). Clinically, a frequent comorbidity between depression and anxiety is observed in adolescents, with evidence suggesting that these conditions may mutually exacerbate each other (12). A prospective study indicated that patients with MDD who also suffer from anxiety are at a higher risk of suicide compared to those experiencing depressive symptoms alone (13). Therefore, early identification of symptoms and the implementation of active treatment models for patients with comorbid depression and anxiety are critical (14). However, family functioning is critically important for adolescent mental health. Murray Bowen’s Family Systems Theory posits that an individual’s emotional distress does not occur alone but stems from the interactions among family members, emotional connections, and intergenerational influences (15). Research has found that an unhealthy family environment, such as parents having mental health problems or experiencing childhood abuse, can exacerbate emotional and behavioral issues in children and adolescents. These adverse factors will significantly increase their risk of developing mental illness (16). Therefore, understanding the association mechanism between childhood abuse and adolescent depression and anxiety is of great significance for formulating targeted intervention measures, improving the function of family systems, and reducing the risk of suicide (17).

Bronfenbrenner’s ecological systems theory highlights the pivotal influence of environmental and familial factors in the development of adolescent mental health. This theoretical framework posits that multiple layers of an individual’s environment, particularly the family unit, exert significant influence on psychological growth and adjustment. Recent empirical studies have demonstrated a robust correlation between childhood abuse and the onset of mental health disorders, including depression, anxiety, and suicidal ideation (18, 19). Childhood abuse is defined as one or more instances of physical, emotional, or sexual abuse, as well as physical or emotional neglect, perpetrated by parents or primary caregivers before the age of 18 (20). Attachment theory focuses on how early relationships affect development. It suggests that these early bonds form the basis for internal working models—mental frameworks that shape how people see themselves and relate to others (21–23). In the context of childhood abuse, children and adolescents frequently develop disorganized attachment patterns, characterized by inconsistent or inadequate caregiving. Such attachment disruptions can have profound and lasting impacts on emotional regulation and psychological functioning. Disorganized attachment is often associated with impaired emotional regulation, which heightens the risk of maladaptive behaviors and increasing susceptibility to mental health disorders such as depression and anxiety. Moreover, attachment disorganization may be linked to dissociative tendencies and attendant problems. This dissociation, a defense mechanism against overwhelming emotions, can further exacerbate psychological distress, including an elevated risk of suicidal ideation (24, 25). Integrating these theoretical perspectives can provide a more detailed and comprehensive understanding of how childhood abuse leads to the development of depression in children and adolescents. Therefore, in-depth research on child abuse is crucial for explaining the mechanisms that lead to the occurrence and development of depressive symptoms in this group. The relevant research conclusions can provide a solid theoretical basis for designing effective prevention and intervention strategies (26).

Traditional studies using regression or structural equation modeling have primarily examined the relationships among depression, anxiety, and childhood maltreatment relationships but are unable to identify symptom-level interactions or dynamic cascades (27, 28). With multimorbidity, symptom networks grow more complex, making simple models insufficient for mapping pathological pathways. To further explore the complex interplay between the dimensions of psychological symptoms and environmental factors, network analysis has emerged as a valuable methodological tool in psychology (29, 30). In this framework, symptoms are represented as nodes, with the relationships between symptoms illustrated by edges (31). The centrality indices of nodes, such as symptom strength and intensity, are particularly crucial for identifying key symptoms, which may inform the selection of intervention targets in clinical practice (30). Consequently, this network-based approach offers a novel perspective on the comorbidity of mental health disorders. The bridge network model is especially effective in revealing the underlying connections among symptoms of comorbid mental disorders and in explaining how external environmental factors influence these symptom interactions (32). In particular, the concept of bridge strength helps identify key bridging nodes that link different symptom clusters, and it illustrates how external factors—such as childhood abuse—can impact specific symptoms across multiple disorders through these nodes (33). In this study, the bridge strength of childhood abuse symptoms clearly shows how environmental influences are directly connected to symptoms of depression and anxiety (34). This is crucial for understanding how the environment interacts with individual symptoms. By clarifying these specific bridging pathways related to childhood abuse, we can improve screening practices, enabling earlier identification of individuals at risk and supporting timely preventive interventions to reduce the long-term psychological effects of abuse (35, 36). The bridge network model, in particular, provides valuable insights into the nature of comorbidities. On one hand, it explains how multiple comorbidities of mental illnesses can arise, while on the other hand, it illustrates how specific symptoms of one disorder may increase the risk of developing another disorder. By delineating the underlying structure of symptom interaction, network analysis offers a deeper understanding of the dynamic relationships among psychological symptoms, enabling more targeted and effective interventions (29).

Currently, numerous studies have utilized network analysis to examine depression and anxiety symptoms in children and adolescents. For instance, a network study on Spanish children and adolescents identified “lfeeling lonely” and “feeling unloved” as central bridge symptoms linking depression and anxiety (37). In addition to general population studies, research has also focused on specific subgroups of children and adolescents, including those with subthreshold depression (38), panic disorder (39), obsessive-compulsive disorder (40), and autism spectrum disorder (41), to investigate the depression–anxiety network. Researchers have further sought to employ network models to elucidate the influence of environmental factors, such as childhood abuse, on depressive symptoms in children and adolescents through specific nodes (42, 43). Given the critical importance and specificity of suicidal ideation within the symptomatology of depression, a Texas−based study explored the direct network relationship between suicidal ideation and depressive symptoms in adolescents (44). However, there is still a significant gap in the current literature. Most network analysis studies involving adolescents have focused on community samples, while there are few studies concentrating on the symptom networks of clinically depressed adolescents with suicidal ideation, especially the interaction of environmental factors (such as childhood abuse) (9). Therefore, this study aims to fill this key gap. It is necessary to investigate clinical samples of adolescents with depression and suicidal ideation, as these adolescents usually exhibit more severe symptoms, a higher incidence of suicidal ideation and more serious social dysfunction compared with the community or school population. It is worth noting that the research results based on community samples may have limitations when generalized to the clinical severe patient population. This is because there may be significant differences in the symptom network structure between the two groups of people (45). For instance, compared with healthy controls, the temporal affective networks of patients with major depressive disorder and mental illness show stronger interconnectivity, which further highlights the essential differences in symptom structure between the clinical and non-clinical populations (46). Therefore, studying this specific high-risk clinical group can provide more precise and clinically relevant insights, inform targeted interventions, and enhance clinical practice. Addressing this gap, our study investigates the network structure of depression, anxiety, and childhood abuse in clinically depressed, suicidal adolescents. Childhood and adolescence are critical periods for mental health development, making symptom-interaction analysis vital for early intervention. By including childhood abuse in our network model, we explore its interplay with depressive/anxiety symptoms, particularly regarding suicidal ideation.

Based on the prior considerations and initial observations, this research is designed to explore three main questions. First, it explores the overall network structure involving symptoms of depression, anxiety, and childhood abuse among clinically depressed adolescents with suicidal ideation. Second, it highlights the specific symptoms occupying central positions within this comorbid symptom network, thereby elucidating potential targets for intervention. Lastly, it examines which symptoms function as critical bridge nodes that interconnect different symptom clusters (e.g., depression, anxiety, and childhood abuse). These bridge nodes may play a role in the spread of psychopathology or highlight the direct impact of environmental factors.

## 2 Methods

### 2.1 Participants and procedure

A cohort comprising 823 adolescent patients, aged between 10 to 18 diagnosed with MDD was recruited from both inpatient and outpatient departments of the Psychiatric Department at the First Affiliated Hospital of Kunming Medical University over the period from June 2021 to December 2023. Diagnosis of MDD was conducted in accordance with DSM-5 criteria by attending psychiatrists or higher-ranking physicians (47). Each participant completed a self-assessment questionnaire, and informed consent was obtained from both participants and their guardians prior to survey administration. Participation was voluntary, and all participants and guardians were informed about the anonymity and confidentiality of the questionnaire. Exclusion criteria encompassed (1): current diagnosis of organic diseases, schizophrenia spectrum disorders, other psychiatric disorders (e.g., neurodevelopmental disorders), bipolar disorder, or psychoactive substance use disorder; (2) incomplete or invalid questionnaire data; (3) inability to understand or cooperate with the completion of questionnaires; (4) not within the specified age range of 10 to 18 years; (5) patients taking multiple psychotropic drugs for treatment.

For this study, the Suicidal Behavior Questionnaire-Revised (SBQ-R) (48), assessed past-year suicidal ideation frequency, specifically querying participants on the frequency of suicidal thoughts in the past year. The response options for this item were: “Never,” “Rarely (1 time),” “Sometimes (2 times),” “Often (3–4 times),” and “Very often (5 or more times).” Participants reporting “never” were excluded. Suicidal ideation occurring 1–2 times was classified as lower frequency, while 3 or more times was categorized as higher frequency. Previous research has demonstrated that this item from the SBQ-R serves as a robust clinical screening tool, aiding in the identification of individuals at elevated risk for suicide and informing subsequent evaluation and intervention strategies (49, 50). The network analysis included 733 MDD-diagnosed children/adolescents with suicidal ideation. Ethical approval was obtained from the Ethics Committee of the First Affiliated Hospital of Kunming Medical University (2021 Ethical Approval L No. 25).

### 2.2 Measures

#### 2.2.1 Childhood trauma

The Child Trauma Questionnaire (CTQ) (51), was utilized in its Chinese version, CTQ-SF Scale, as adapted by FU-wenqing (52), to assess childhood trauma. This instrument comprises 28 items rated on a five-point scale ranging from 1 (“never”) to 5 (“always”), where higher scores indicate greater severity of childhood trauma. The internal consistency of the scale, assessed by Cronbach’s α coefficient in this study, was 0.82, indicating good reliability.

#### 2.2.2 Depression

The Patient Health Questionnaire (PHQ-9) (53), a nine-item tool using a four-point Likert scale, assessed depressive symptoms in children and adolescents. This clinically validated measure demonstrated excellent internal consistency (Cronbach’s α = 0.86) in our study (54).

#### 2.2.3 Anxiety

The Generalized Anxiety Disorder Scale (GAD-7) (55), a seven-item instrument using a four-point Likert scale, assessed participants’ anxiety symptoms. This scale has demonstrated strong psychometric properties and effectiveness for screening anxiety in Chinese adolescents (56). In our study, the GAD-7 showed excellent internal consistency (Cronbach’s α = 0.89).

### 2.3 Analysis

Descriptive statistics were computed using SPSS, and the network structure estimation was conducted in R (version 4.4.1) employing several dedicated packages, namely *bruceR*, *qgraph*, *networktools*, *bootnet*, and *mgm*.

### 2.4 Network construction

#### 2.4.1 Network estimation

Network analyses were performed using R 4.4.1. Considering our sample size, data type, and research objectives, we estimated network models and calculated connection weights using the graphical LASSO method with EBIC model selection at first, implemented through the estimateNetwork function with EBICglasso option (57). In the resulting networks, nodes represent variables connected by edges whose thickness reflects connection strength (30). Node predictability (how well each node is predicted by others) was assessed using the mgm package (45).

#### 2.4.2 Centrality and bridge estimation

In the exploration of network structure, three key centrality indicators are considered: strength, closeness, and betweenness (58). Centrality indices for each node were calculated using the centralityPlot function from the graph package in R (59).

Strength refers to the sum of the absolute values of the weights of the edges connected to a node. Prior research has highlighted the relevance of node strength in the study of psychopathology, noting its greater stability compared to other centrality measures like closeness and betweenness (31, 60).

A bridge analysis was conducted to identify key pathways linking depressive symptoms and environmental factors. Bridge nodes, critical connections between network domains, were assessed using bridge strength (networktools package in R). Bridges are shared dimensions that facilitate symptom transmission across domains (61). Bridge strength, the most effective index (62), was estimated with an 80th percentile cutoff (61), revealing influential pathways and network dynamics.

#### 2.4.3 Network accuracy and stability

Given the influence of sampling variability on the estimation of network models, we employed the bootstrap method to assess the accuracy and stability of both edge weights and centrality measures. This was accomplished using the bootnet package in R. To evaluate the accuracy of the edge weights, we calculated the 95% confidence intervals (CI) for the edge-weight bootstrap. For assessing the stability of centrality measures, we utilized the centrality stability coefficient (CS coefficient). This coefficient serves as a reference index for determining the robustness of the centrality measures against sampling variability.

## 3 Results

### 3.1 Descriptive statistics

The study enrolled a total of 733 subjects (N = 733), with a mean age of 14.81 years ± standard deviation of 1.63 years. Among the 733 individuals in this cohort, 548 (74.8%) were of Han ethnicity. Among the 548 people in this cohort, 74.8% are of Han ethnicity. In addition, 536 participants (73.1%) were urban residents, and 341 participants (46.5%) were only children. Detailed demographic characteristics are presented in **Table 1**.

**Table 1:** Demographic characteristics of the sample (N = 733).

Variable	Mean (SD) or N (%)
Age (years)	14.81 (1.63)
Sex	
Male	190 (25.9%)
Female	543 (74.1%)
Only child: Yes	341 (46.5%)
Current residence region	
City	536 (73.1%)
Rural	197 (26.9%)
Ethnicity	
Han	548 (74.8%)
Bai	117 (16.0%)
Yi	37 (5.0%)
Hui	9 (1.2%)
Others	22 (3.0%)
Live with mother: Yes	631 (86.1%)
Live with father: Yes	490 (66.8%)
Frequency of suicidal ideation	
1 time	94 (12.8%)
2 times	162 (22.1%)
3–4 times	182 (24.8%)
5 or more times	295 (40.2%)

### 3.2 Network structure

**Figure 1:**
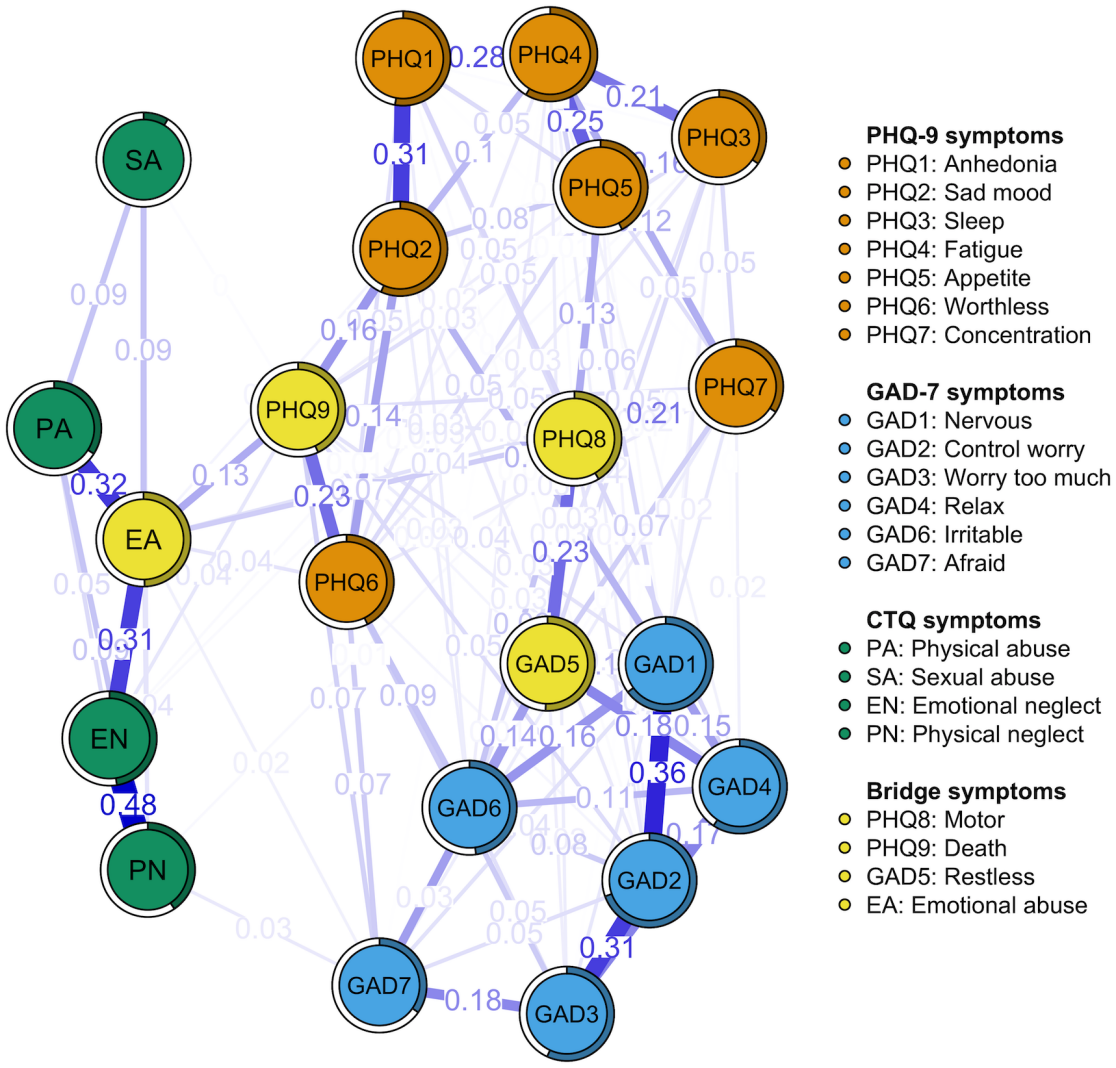
The network displaying the relationship between depression, anxiety, and childhood abuse symptoms. Nodes of different colors represent distinct network communities. Specifically, yellow nodes denote bridge nodes. The specific meanings and values of each node are shown in **Table 2**. Nodes are connected by edges, with thicker edges indicating stronger connections between symptom nodes.

**Figure 1** illustrates the network structure of depression, anxiety, and childhood abuse in Depressed Adolescents with suicidal ideation. Out of 210 possible edges, 109 (51.90%) non-zero edges were observed, with an average weight of 0.04. The network demonstrated overall positive correlations among symptoms. Predictability of the symptoms is represented in **Figure 1** as a ring pie chart, with an average predictability score of 0.46. This indicates that adjacent nodes in the model can explain 46% of the variance of each node on average.

In the network model, as depicted in **Figure 1**, the nodes representing “emotional neglect” (EN) and “physical neglect” (PN) exhibited the strongest direct relationship within the childhood abuse symptom group (r = 0.48). This was followed by the association between “emotional abuse” (EA) and “physical abuse” (PA) (r = 0.32), and between EA and EN (r = 0.31). Meanwhile, within the depressive symptom group, the node representing “anhedonia” (PHQ1) and the node “sad mood” (PHQ2) were most directly connected (r = 0.31). This was followed by connections between the PHQ1 and the “fatigue” node (PHQ4) (r = 0.28), the PHQ4 and the “appetite” node (PHQ5) (r = 0.25), and the “worthless” node (PHQ6) and the “Death” node (PHQ9) (r = 0.23). In the anxiety symptom group, the connection between node “nervous” (GAD1) and node “Control worry” (GAD2) (r = 0.36) is the most direct. This is followed by the connection between node GAD2 and node “worry too much” (GAD3) (r = 0.31), and the connection between GAD3 and node “relax” (GAD4) (r = 0.19). Furthermore, there are numerous interconnections between symptoms across the three communities. For instance, the node “restless” (GAD5) is most closely associated with the node “motor” (PHQ8) (r = 0.23). Additionally, there is a notable connection between PHQ9 and EA (r = 0.13). **Supplementary Table 1** details all edge weights within the network.

### 3.3 Network central and bridge symptoms

**Figure 2:**
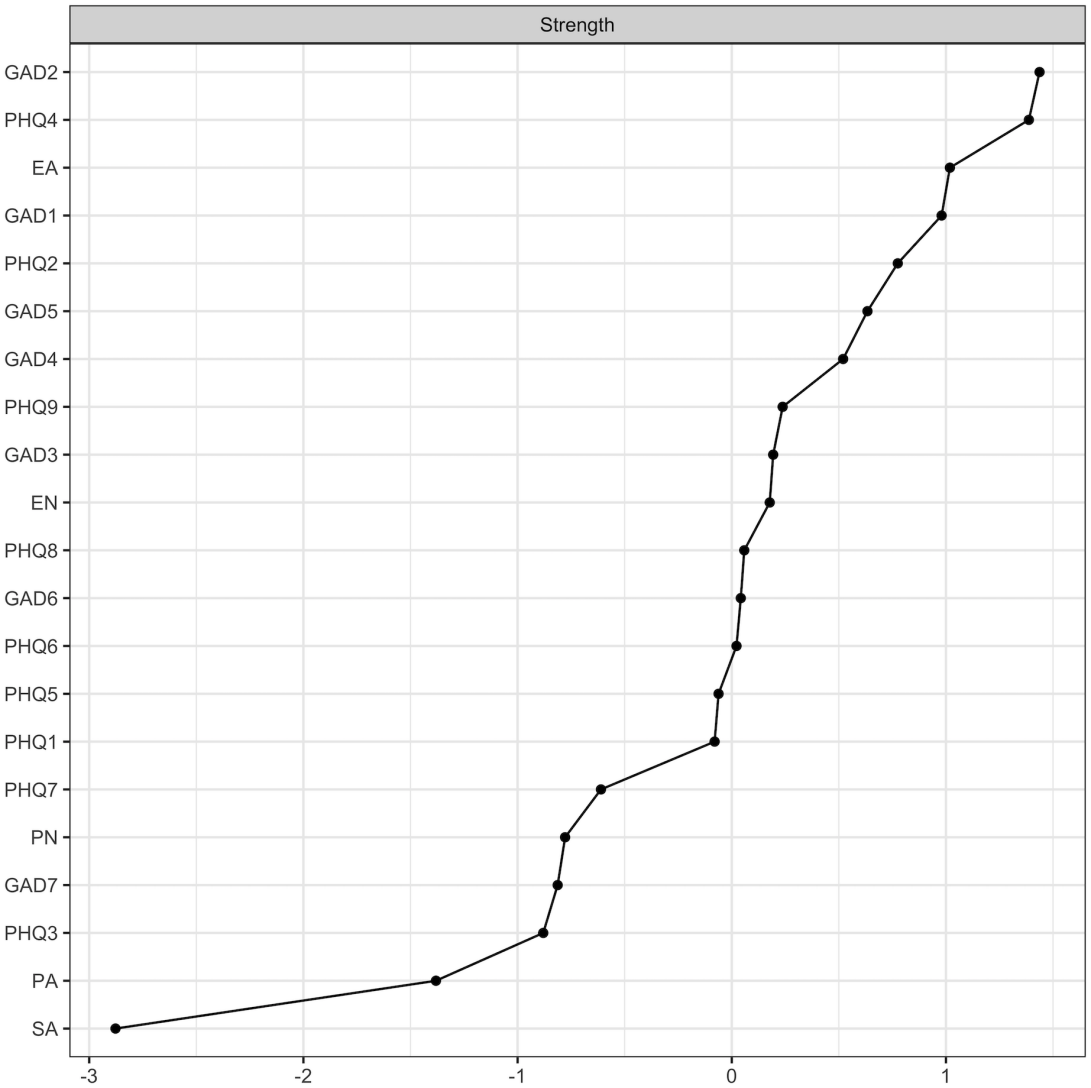
Node strength centrality estimates of the present network.

The intensity of nodes in children and adolescents with MDD and suicidal ideation is depicted in **Figure 2**. Among these nodes, GAD2 emerges as the most robust, with nodes PHQ4 and EA also demonstrating statistically significant strength compared to most other nodes in the network. This indicates that these nodes are more central to the overall network structure. Additional centrality indicators can be found in **Supplementary Figure 1**.

**Figure 3:**
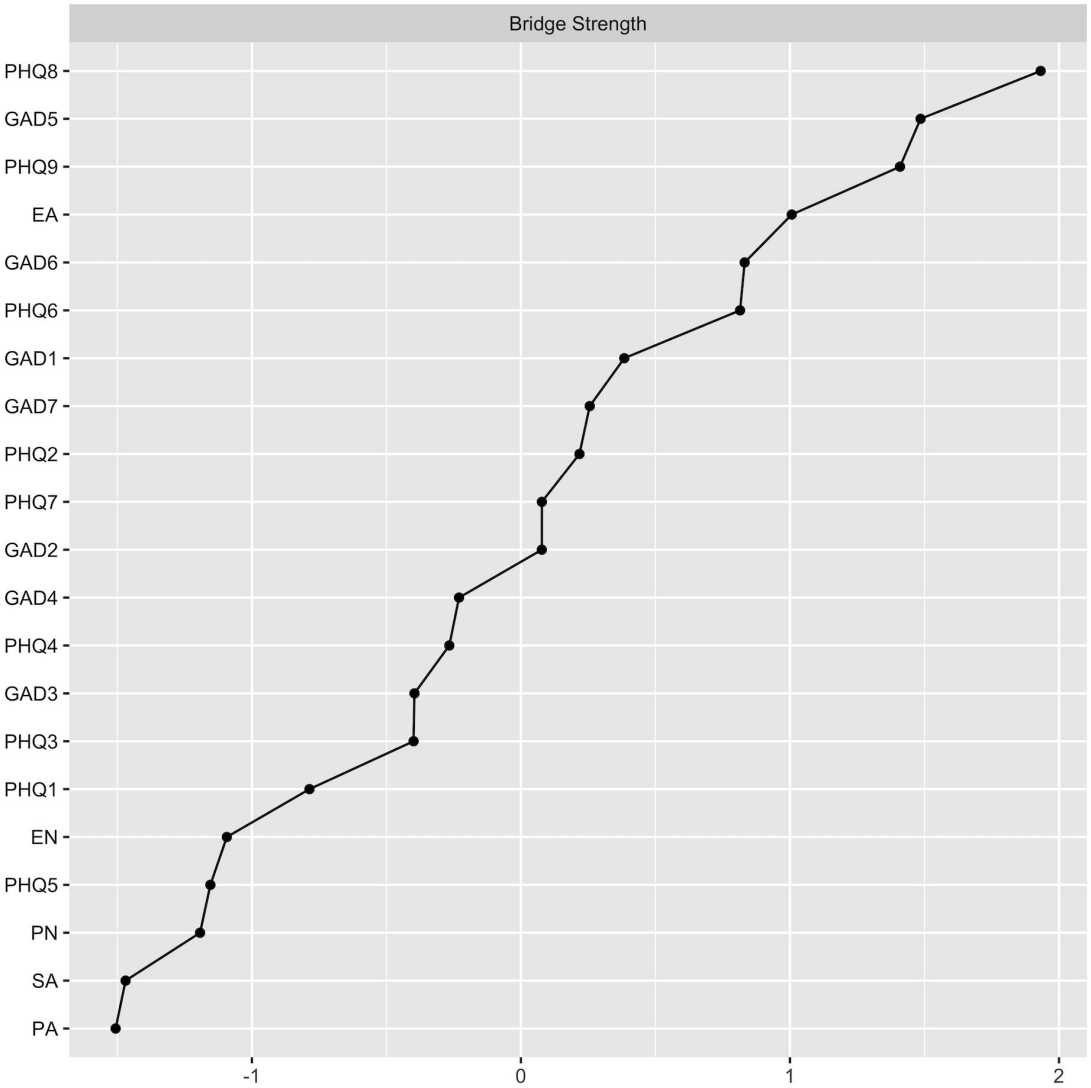
Bridge strength of each node in the present network.

To gain a deeper understanding of the relationships between different symptom communities, the bridge strength of each node was calculated, resulting in the bridge network illustrated in **Figure 1**. According to the bridge strength analysis shown in **Figure 3**, the nodes PHQ8, PHQ9, GAD5, and EA exhibit higher bridge strength compared to most other nodes. This signifies that these four nodes serve as critical bridging symptoms, linking various symptom communities within the network. **Table 2** presents the raw values for the strength of all symptoms and their respective bridge strengths. We estimated networks for the Han and urban subgroups, and their network structurekey, central symptoms and bridge symptoms were largely consistent with the overall sample (**Supplementary Figures 5**, **6**).

**Table 2:** Descriptive statistics of depression, anxiety, and childhood abuse symptoms in depressed adolescents.

Item	Item abbreviation	Mean (SD)	Strength	Bridge strength	Predictability
PHQ1	Anhedonia	2.41 (0.73)	0.83	0.09	0.53
PHQ2	Sad mood	2.35 (0.74)	1.02	0.22	0.57
PHQ3	Sleep	2.20 (0.94)	0.66	0.14	0.35
PHQ4	Fatigue	2.39 (0.79)	1.15	0.16	0.59
PHQ5	Appetite	2.17 (0.93)	0.84	0.04	0.42
PHQ6	Worthless	2.27 (0.89)	0.86	0.29	0.44
PHQ7	Concentration	1.88 (1.07)	0.72	0.20	0.34
PHQ8	Motor	1.74 (1.07)	0.86	0.43	0.41
PHQ9	Death	1.94 (0.97)	0.90	0.37	0.43
GAD1	Nervous	2.30 (0.85)	1.07	0.24	0.65
GAD2	Control worry	2.20 (0.92)	1.17	0.20	0.70
GAD3	Worry too much	2.13 (0.90)	0.89	0.14	0.57
GAD4	Relax	2.11 (0.90)	0.96	0.16	0.60
GAD5	Restless	1.73 (1.01)	0.99	0.38	0.51
GAD6	Irritable	2.36 (0.82)	0.86	0.29	0.48
GAD7	Afraid	1.62 (1.08)	0.67	0.22	0.35
EA	Emotional abuse	11.88 (4.87)	1.07	0.32	0.50
PA	Physical abuse	7.62 (3.42)	0.55	0.00	0.34
SA	Sexual abuse	5.53 (1.73)	0.22	0.00	0.09
EN	Emotional neglect	15.85 (4.97)	0.89	0.05	0.48
PN	Physical neglect	9.46 (3.50)	0.68	0.04	0.41

### 3.4 Network accuracy and stability

**Figure 4:**
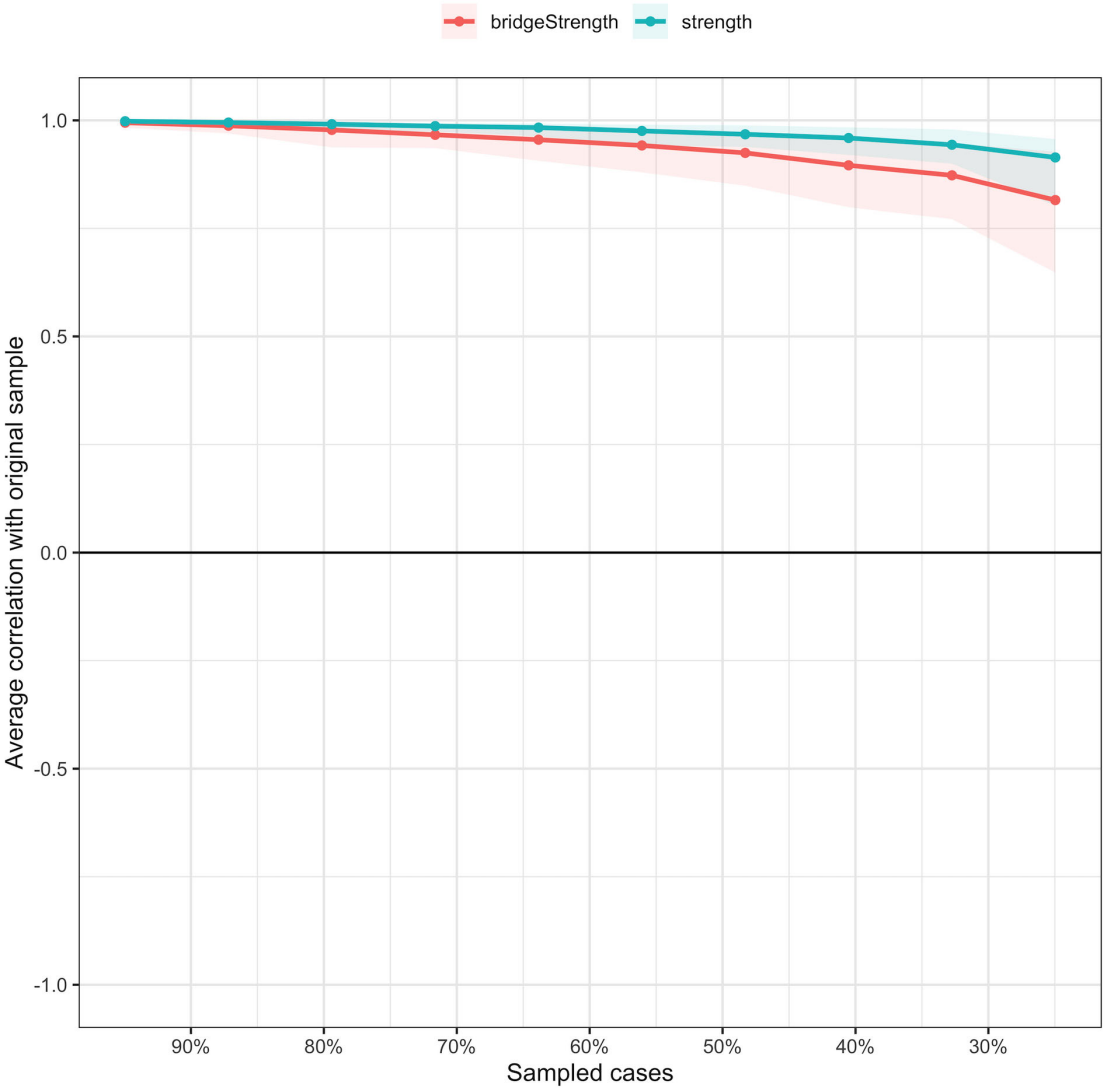
The stability of centrality and bridge centrality indices using case-dropping bootstrap.

As illustrated in **Supplementary Figure 2**, the bootstrap 95% CI is narrow, indicating that the estimates of edge weights are both accurate and stable. Results from the bootstrap difference test for edge weights (**Supplementary Figure 3**) reveal that the strongest edges in the network are found between items from the childhood abuse scale. **Supplementary Figure 4** shows the difference test of node strength. The stability of the CS for node strength and bridge strength is reflected in a CS coefficient of 0.75 and 0.67, indicating that these estimates are sufficiently stable (see **Figure 4**). Additionally, the CS coefficients for the remaining centrality indicators are all greater than 0.25 (see **Supplementary Figure 7**). Additionally, the subgroup networks showed good stability (**Supplementary Figures 8**, **9**). Given that the estimates of node strength are more reliable, the interpretation of the results in this study primarily focuses on node strength.

## 4 Discussion

In contrast to many studies that utilize samples from adult populations within communities, this research adopts a network analysis approach to investigate the relationships between childhood maltreatment, depression, and anxiety among outpatient or inpatient pediatric and adolescent patients with suicidal ideation. This methodological approach is relatively uncommon in existing research. By employing this method, we aim to elucidate the complex interconnections among various community clusters, as well as the intricate interactions and associations between environmental factors and symptoms. Specifically (1): Among the symptoms across the three communities, the node GAD5 exhibits the strongest connection with the node PHQ8; (2) The nodes GAD2, PHQ4, and EA are the most central symptoms within the comorbid network model; (3) The node with the highest bridging strength in the comorbid network is PHQ8, which will be further discussed in detail below.

### 4.1 Central nodes and edges in the depression-anxiety-childhood abuse network

We have found that depression and anxiety symptoms in adolescents with suicidal ideation are highly interconnected and form a cluster. Within the depression and anxiety network, we observed that “control worry” and “fatigue” show high strength, making them core symptoms of the network, which is consistent with previous research conducted in child and adolescent populations (63, 64). Fatigue not only serves as one of the important symptoms for the diagnostic criteria of depression (47), but has also been mentioned in numerous studies of depressive symptoms. For instance, a depression symptom network study involving 3,463 outpatients with depression in the United States revealed that lack of energy has the strongest node centrality (65). Another survey conducted among college students demonstrated that fatigue is associated with more severe depressive symptoms and a higher risk of suicidal and anxiety tendencies (66).

Empirical evidence indicates that adolescents with depression exhibit a distinct depressive symptom profile characterized by pronounced fatigue, in contrast to adult populations (67). From a developmental psychopathology perspective, this phenomenon may be attributed to the interplay of multiple biological and psychosocial factors, including pubertal hormonal fluctuations and circadian rhythm dysregulation, which collectively predispose adolescents to increased vulnerability to persistent fatigue and energy depletion (68, 69). This finding prompts us to investigate the sensation of fatigue in depressive symptoms, which may uncover the complex relationships between depression, anxiety, and suicide. Interestingly, related neuroimaging studies have also shown that reduced neuronal activity in relevant brain regions of individuals with depression may explain the symptom of fatigue (70).

Furthermore, we found that “control worry” consistently exhibits high node centrality across almost all network models studying depressive and anxiety symptoms. Despite using a sample of patients with depression in this study, we obtained similar results. Childhood and adolescence are critical periods for brain structural changes and development (71), potentially related to cognitive formation. According to the cognitive theory of suicide (72), the occurrence of suicidal behavior is associated with negative cognitions among individuals with depression. Children and adolescents with depression often have negative self-cognitions, manifested as pessimistic expectations about themselves, their environment, and the future, which may lead to persistent tension and worry, and subsequently, suicidal ideation.

Within the overarching framework of the depression-anxiety-childhood abuse network, EA emerges as a node with greater strength, alongside “control worry” and “fatigue”, echoing analogous findings in prior research on childhood trauma networks (42). A deeper exploration reveals that the most pronounced edges are predominantly observed among the various forms of childhood maltreatment, with a notable emphasis on EA. Our findings well with those reported in earlier studies (73). Indeed, previous scholars have documented the frequent coexistence of psychological abuse with PA (74). This concurrence supports our research conclusions and further substantiates the intricate interplay between diverse manifestations of childhood maltreatment. We postulate that, within the familial sphere, the occurrence of childhood maltreatment, including sexual abuse, may exacerbate familial conflicts. Both the familial milieu and maltreatment experiences have the potential to impact children’s depressive and anxiety symptoms (75). A comprehensive meta-analysis indicates that individuals exposed to childhood maltreatment exhibit a twofold increased risk of recurrent depressive episodes compared to those without such histories (76). Hence, irrespective of the maltreatment type, any such experience poses a significant threat to the mental health of children and adolescents.

Conversely, in the realm of psychiatric symptoms, the strongest edges are predominantly found within the respective communities of depressive and anxiety symptoms, rather than between them, aligning with extensive prior research. Across these investigations, we consistently identify the most robust edges within depressive or anxiety symptom clusters, using PHQ-9 and GAD-7 scales to identify connections such as GAD1-GAD2, GAD2-GAD3, and PHQ1-PHQ2 (77, 78). However, differences in the pattern of strongest edges become evident when our findings are compared with certain studies (79), potentially attributed to our focus on healthcare workers. In the post-pandemic era, the frequent revisions of infection control protocols and guidelines have introduced an element of uncertainty. Compared with the general populace, healthcare workers confront unique challenges, including the implementation of infectious disease prevention measures and the management of critically ill patients (80), which may manifest in distinct depressive and anxiety profiles.

Nonetheless, among the top ten strongest edges identified, only one bridges the depressive and anxiety symptom communities, specifically PHQ8 and GAD5, resonating with prior research (77). This cross-community edge underscores the potential of these symptoms as candidate bridge symptoms within the entire network. From a clinical standpoint, the strong association between the “Motor” and “Restless” nodes suggests that interventions targeting the somatic manifestations of internal distress, particularly psychomotor agitation, may simultaneously alleviate symptoms of both depression and anxiety.

### 4.2 Bridge symptoms in the depression-anxiety-childhood abuse network

Regarding the connection between adverse childhood experiences and depressive and anxiety symptoms in adolescents, we found nodes within the communities of childhood trauma, depression, and anxiety that collectively serve as crucial bridges. Specifically, PHQ8, PHQ9, GAD5, and EA exhibit the highest bridge strength. As illustrated in **Figure 1**, PHQ9 and EA link the two communities of depression and childhood maltreatment (81). Previous researchers posited that emotional abuse constitutes a persistent, repetitive, and inappropriate emotional response to children’s emotional expressions and their accompanying behaviors.

According to the cumulative risk hypothesis, negative events in early childhood, such as maltreatment, may have a cumulative effect, leading to increased internalizing and externalizing behavior problems in the future (82), which may ultimately result in suicidal ideation. The despair theory is often utilized by researchers to explain the linkage between negative events, emotions, and suicide (83). On one hand, individuals with depressive disorders may develop suicidal ideation due to cognitive changes that lead to feelings of hopelessness and diminished Reasons for Living (84). On the other hand, individual negative life events, such as emotional abuse, can also induce feelings of despair. Specifically, despair mediates the relationship between emotional abuse and depressive symptoms, and negative life events combined with negative cognition can predict despair and depressive symptoms (85), potentially leading to the emergence of suicidal ideation. Our network analysis identified cognitive-affective symptoms as central mediators that transmit the impact of emotional abuse toward suicidal ideation, highlighting a potential psychopathological pathway underlying this association.

In China, parents often see their children as “private property” and believe they have the right to beat their children when they try to educate and “regulate” them (86). Therefore, it is necessary to fundamentally change this concept and phenomenon. In addition, the incidence of child abuse can be reduced to some extent through training (87). Target adolescents exposed to childhood abuse to develop positive personality traits and improve emotional regulation skills (88). This measure is essential for children who have already been diagnosed with mdd. It is important to note that when children experience more abuse, even more positive experiences (such as social support, peer care, etc.) do not significantly improve the exposure of adverse experiences to mental health problems (89), so it is very possible to prevent childhood abuse. However, there are documented reasons why child abuse has not yet been recognized as a social problem worthy of public attention in China (90), and this phenomenon needs to be greatly changed. On the one hand, correct guidance of public opinion can be established through media and Internet to reduce the occurrence of childhood abuse. On the other hand, strengthen the formulation of relevant laws and regulations, restrict the parenting behavior, and maximize the protection of children and adolescents from domestic abuse.

This study identified key bridge nodes linking depression, anxiety, and childhood abuse through bridge network analysis, offering a new perspective for clinical identification and intervention. PHQ8, GAD5, and EA emerged as important hubs connecting different psychological problems. In outpatient screening, elevated scores on these bridge nodes may indicate the presence of comorbid depression and anxiety as well as a history of childhood abuse, warranting further assessment of suicide risk (91). In terms of treatment, for individuals with high scores on bridge nodes such as EA and GAD5, different strategies may be applied. For example, targeting restlessness (GAD-5) directly may be effective, as studies have shown that early improvement in restlessness is associated with remission in major depression (92). For patients with high EA scores, it is essential to assess for a potential history of childhood trauma. Interventions focusing on trauma and family functioning may help reduce the mutual reinforcement between depression and anxiety, potentially lowering overall suicide risk (93).

### 4.3 Strengths, limitations and future direction

The strength of this study lies in its focus on clinically diagnosed depressive populations, where it identifies children with high-risk suicidal ideation and incorporates childhood maltreatment, along with depressive and anxiety symptoms, into the model analysis.

However, the study also has certain limitations. Firstly, it only includes childhood maltreatment as an environmental factor in the network model, neglecting other risk factors. Confounding variables (e.g., socioeconomic status, pharmacologic treatment, or trauma recency) were not controlled, which may subtly influence certain symptom-to-symptom associations. Nonetheless, we believe the core findings—such as the strong bridge connections between abuse and symptoms—are robust. We recommend future studies include more risk factors and control for confounders to further validate these associations. Secondly, as a cross-sectional study, we cannot establish the temporal sequence and causal relationship between childhood maltreatment experiences and depressive/anxiety symptoms. Results from a single time point are inadequate for understanding the entire process. Future studies should conduct dynamic observations of variables, with longitudinal data and cross-lagged network models potentially providing better explanations. Thirdly, The SBQ-R was used for measurement. However, the information obtained from individual item scores is relatively limited, as the screening was based solely on questions related to suicidal ideation. Future research could further investigate the causal inference of abuse and depressive symptoms on suicidal ideation within network structures (94). We also considered combining the SBQ-R with the Columbia Suicide Severity Rating Scale to assess different dimensions of suicidality. Lastly, as our sample was drawn from a single hospital, the generalizability of our findings is limited to similar clinical populations. Future studies will aim to recruit adolescents with depression from multiple hospitals and diverse regional backgrounds.

## 5 Conclusion

In conclusion, this study employed symptom network analysis to examine interactions between depression and anxiety symptoms and childhood adversity in suicidal adolescents with depressive disorders. The network revealed strong depression-anxiety comorbidity, with “control worry” and “fatigue” as central nodes. Bridge centrality identified transdiagnostic bridge symptoms (e.g., “motor”, “death”), which may amplify suicide risk through cognitive-affective pathways. Childhood maltreatment subtypes showed strong intra-correlations, with EA directly linked to “death”, suggesting it indirectly fuels suicidal ideation through negative schemas (e.g., hopelessness). To safeguard adolescent mental health and reduce suicide risk, interventions are needed at family, psychological, and policy levels. Our findings highlight the importance of a holistic approach, considering the complex links between childhood abuse, suicidality, and psychiatric symptoms.

## Data Availability

The raw data supporting the conclusions of this article will be made available by the authors, without undue reservation.

## References

[1] World Health Organization. Depressive disorder (depression)(2023). Available online at: https://www.who.int/news-room/fact-sheets/detail/depression (Accessed February 13, 2025).

[2] Lu J Xu X Huang Y Li T Ma C Xu G. Prevalence of depressive disorders and treatment in China: a cross-sectional epidemiological study. Lancet Psychiatry. (2021) 8:981–90. doi: 10.1016/S2215-0366(21)00251-0, PMID: 34559991

[3] Mullen S. Major depressive disorder in children and adolescents. Ment Health Clin. (2018) 8:275–83. doi: 10.9740/mhc.2018.11.275, PMID: 30397569 PMC6213890

[4] Xiaolan F Kan Z Xuefeng C Zhiyan C. Report on National Mental Health Development in China (2020-2021). China: Social Sciences Academic Press (2023). 319 p.

[5] Racine N McArthur BA Cooke JE Eirich R Zhu J Madigan S. Global prevalence of depressive and anxiety symptoms in children and adolescents during COVID-19: a meta-analysis. JAMA Pediatr. (2021) 175:1142. doi: 10.1001/jamapediatrics.2021.2482, PMID: 34369987 PMC8353576

[6] Kang C Zheng Y Yang L Wang X Zhao N Guan TF. Prevalence, risk factors and clinical correlates of suicidal ideation in adolescent patients with depression in a large sample of chinese. J Affect Disord. (2021) 290:272–8. doi: 10.1016/j.jad.2021.04.073, PMID: 34015621

[7] Cantor N Kingsbury M Warner E Landry H Clayborne Z Islam R. Young adult outcomes associated with adolescent suicidality: a meta-analysis. Pediatrics. (2023) 151:e2022058113. doi: 10.1542/peds.2022-058113, PMID: 36810672

[8] Walsh EH McMahon J Herring MP. Research review: the effect of school-based suicide prevention on suicidal ideation and suicide attempts and the role of intervention and contextual factors among adolescents: a meta-analysis and meta-regression. J Child Psychol Psychiatry. (2022) 63:836–45. doi: 10.1111/jcpp.13598, PMID: 35289410 PMC9544521

[9] Gijzen MWM Rasing SPA Creemers DHM Smit F Engels RCME De Beurs D. Suicide ideation as a symptom of adolescent depression. a network analysis. J Affect Disord. (2021) 278:68–77. doi: 10.1016/j.jad.2020.09.029, PMID: 32956963

[10] Chang Q Shi Y Yao S Ban X Cai Z. Prevalence of suicidal ideation, suicide plans, and suicide attempts among children and adolescents under 18 years of age in mainland China: a systematic review and meta-analysis. Trauma Violence Abuse. (2024) 25:2090–102. doi: 10.1177/15248380231205828, PMID: 37902618

[11] Soylu N Tanelİ Y Tanelİ S. Investigation of social, emotional, and cognitive factors with effect on suicidal behaviour in adolescents with depression. Nöro Psikiyatri Arş. (2013) 50:352–9. doi: 10.4274/Npa.y6531, PMID: 28360569 PMC5363428

[12] Cummings CM Caporino NE Kendall PC. Comorbidity of anxiety and depression in children and adolescents: 20 years after. Psychol Bull. (2014) 140:816–45. doi: 10.1037/a0034733, PMID: 24219155 PMC4006306

[13] Bolton JM Pagura J Enns MW Grant B Sareen J. A population-based longitudinal study of risk factors for suicide attempts in major depressive disorder. J Psychiatr Res. (2010) 44:817–26. doi: 10.1016/j.jpsychires.2010.01.003, PMID: 20122697 PMC2888712

[14] Rajkumar RP. Comorbid depression and anxiety: integration of insights from attachment theory and cognitive neuroscience, and their implications for research and treatment. Front Behav Neurosci. (2022) 16:1104928. doi: 10.3389/fnbeh.2022.1104928, PMID: 36620859 PMC9811005

[15] Bowen M. The use of family theory in clinical practice. Compr Psychiatry. (1966) 7:345–74. doi: 10.1016/S0010-440X(66)80065-2, PMID: 5922263

[16] Gonzalez A Boyle MH Kyu HH Georgiades K Duncan L MacMillan HL. Childhood and family influences on depression, chronic physical conditions, and their comorbidity: findings from the ontario child health study. J Psychiatr Res. (2012) 46:1475–82. doi: 10.1016/j.jpsychires.2012.08.004, PMID: 22959202

[17] Chaplo SD Shepard Abdulahad LD Keeshin BR. Utilizing screening as a trauma-responsive approach in pediatric health care settings. Curr Probl Pediatr Adolesc Health Care. (2024) 54:101548. doi: 10.1016/j.cppeds.2023.101548, PMID: 38336539

[18] Chen X Jiang L Liu Y Ran H Yang R Xu X. Childhood maltreatment and suicidal ideation in chinese children and adolescents: the mediation of resilience. PeerJ. (2021) 9:e11758. doi: 10.7717/peerj.11758, PMID: 34277155 PMC8269734

[19] Sahle BW Reavley NJ Morgan AJ Yap MBH Reupert A Jorm AF. How much do adverse childhood experiences contribute to adolescent anxiety and depression symptoms? Evidence from the longitudinal study of Australian children. BMC Psychiatry. (2024) 24:289. doi: 10.1186/s12888-024-05752-w, PMID: 38632617 PMC11022337

[20] Li S Zhao F Yu G. A meta-analysis of childhood maltreatment and intimate partner violence perpetration. Aggress Violent Behav. (2020) 50:101362. doi: 10.1016/j.avb.2019.101362

[21] Ainsworth MS. Infant–mother attachment. Am Psychol. (1979) 34:932–7. doi: 10.1037/0003-066X.34.10.932 517843

[22] Barbour RF. Attachment and loss. Vol. 1. Attachment. By john bowlby. London: the hogarth press and institute of psycho-analysis. 1969. Pp. 428. Price 63 *s* . Br J Psychiatry. (1970) 116:102–3. doi: 10.1192/bjp.116.530.102

[23] Crittenden PM. Social networks, quality of child rearing, and child development. Child Dev. (1985) 56:1299. doi: 10.2307/1130245

[24] Crittenden PM Ainsworth MDS. Child maltreatment and attachment theory. In: Cicchetti D Carlson V, editors. Child Maltreatment. Cambridge: Cambridge University Press (1989). p. 432–63. doi: 10.1017/CBO9780511665707.015

[25] Sroufe LA. Attachment and development: A prospective, longitudinal study from birth to adulthood. Attach Hum Dev. (2005) 7:349–67. doi: 10.1080/14616730500365928, PMID: 16332580

[26] Kang J. The impact of childhood maltreatment timing on adolescents’ depression and anxiety. Fam Soc: J Contemp Soc Serv. (2024) 1. doi: 10.1177/10443894241272258

[27] Zhang Y Liao H Gu J Wang J. Anxiety and depression related to childhood maltreatment in teenagers: comparing multiple individual risk model, cumulative risk model and latent profile analysis. Child Abuse Negl. (2022) 128:105630. doi: 10.1016/j.chiabu.2022.105630, PMID: 35413546

[28] Reis DL Ribeiro M Couto I Maia N Bonavides D Botelho AC. Correlations between childhood maltreatment and anxiety and depressive symptoms and risk behaviors in school adolescents. Trends Psychiatry Psychother. (2024) 46:e20210456. doi: 10.47626/2237-6089-2021-0456, PMID: 36645004 PMC11140768

[29] Cramer AOJ Waldorp LJ van der Maas HLJ Borsboom D. Comorbidity: a network perspective. Behav Brain Sci. (2010) 33:137–50. doi: 10.1017/S0140525X09991567, PMID: 20584369

[30] Borsboom D Cramer AOJ. Network analysis: an integrative approach to the structure of psychopathology. Annu Rev Clin Psychol. (2013) 9:91–121. doi: 10.1146/annurev-clinpsy-050212-185608, PMID: 23537483

[31] Epskamp S Borsboom D Fried EI. Estimating psychological networks and their accuracy: a tutorial paper. Behav Res Methods. (2018) 50:195–212. doi: 10.3758/s13428-017-0862-1, PMID: 28342071 PMC5809547

[32] Briganti G Scutari M Epskamp S Borsboom D Hoekstra RHA Golino HF. Network analysis: an overview for mental health research. Int J Methods Psychiatr Res. (2024) 33(4):e2034. doi: 10.1002/mpr.2034, PMID: 39543824 PMC11564129

[33] Guloksuz S Van Nierop M Bak M De Graaf R Ten Have M Van Dorsselaer S. Exposure to environmental factors increases connectivity between symptom domains in the psychopathology network. BMC Psychiatry. (2016) 16:223. doi: 10.1186/s12888-016-0935-1, PMID: 27391407 PMC4939022

[34] Conway JR. Assessing anxiety and depression in young populations: an inventory of environmental risk factors and adverse childhood events. CAND J. (2020) 27:20–2. doi: 10.54434/candj.49

[35] Dodge KA Benjamin Goodman W Bai Y Murphy RA O’Donnell K. Maximizing the return on investment in early childhood home visiting through enhanced eligibility screening. Child Abuse Negl. (2021) 122:105339. doi: 10.1016/j.chiabu.2021.105339, PMID: 34560398 PMC9623423

[36] Hahn H Oransky M Epstein C Smith Stover C Marans S. Findings of an early intervention to address children’s traumatic stress implemented in the child advocacy center setting following sexual abuse. J Child Adolesc Trauma. (2016) 9:55–66. doi: 10.1007/s40653-015-0059-7

[37] Sánchez Hernández MO Carrasco MA Holgado-Tello FP. Anxiety and depression symptoms in spanish children and adolescents: an exploration of comorbidity from the network perspective. Child Psychiatry Hum Dev. (2023) 54:736–49. doi: 10.1007/s10578-021-01286-4, PMID: 34797464 PMC10140092

[38] Fan P Wang T Wang J Wang J. Network analysis of comorbid depression and anxiety and their associations with response style among adolescents with subthreshold depression. Curr Psychol. (2024) 43:8665–74. doi: 10.1007/s12144-023-04992-5

[39] Cha EJ Hong S Park D-H Ryu S-H Ha JH Jeon HJ. A network analysis of panic symptoms in relation to depression and anxiety sensitivity in patients with panic disorder. J Affect Disord. (2022) 308:134–40. doi: 10.1016/j.jad.2022.04.062, PMID: 35429524

[40] Cervin M Lázaro L Martínez-González AE Piqueras JA Rodríguez-Jiménez T Godoy A. Obsessive-compulsive symptoms and their links to depression and anxiety in clinic- and community-based pediatric samples: a network analysis. J Affect Disord. (2020) 271:9–18. doi: 10.1016/j.jad.2020.03.090, PMID: 32312700

[41] Montazeri F De Bildt A Dekker V Anderson GM. Network analysis of anxiety in the autism realm. J Autism Dev Disord. (2019) 49:2219–30. doi: 10.1007/s10803-018-3474-4, PMID: 29383649

[42] Guo W Zhao Y Chen H Liu J Chen X Tang H. The bridge symptoms of childhood trauma, sleep disorder and depressive symptoms: a network analysis. Child Adolesc Psychiatry Ment Health. (2023) 17:88. doi: 10.1186/s13034-023-00635-6, PMID: 37403102 PMC10320961

[43] Wang K Hu Y He Q Xu F Wu YJ Yang Y. Network analysis links adolescent depression with childhood, peer, and family risk environment factors. J Affect Disord. (2023) 330:165–72. doi: 10.1016/j.jad.2023.02.103, PMID: 36828149

[44] Guzick A Storch EA Smárason O Minhajuddin A Drummond K Riddle D. Psychometric properties of the GAD-7 and PROMIS-anxiety-4a among youth with depression and suicidality: results from the texas youth depression and suicide research network. J Psychiatr Res. (2024) 170:237–44. doi: 10.1016/j.jpsychires.2023.12.033, PMID: 38169247

[45] Haslbeck JMB Fried EI. How predictable are symptoms in psychopathological networks? A reanalysis of 18 published datasets. Psychol Med. (2017) 47:2767–76. doi: 10.1017/s0033291717001258, PMID: 28625186

[46] Fried EI Van Borkulo CD Cramer AOJ Boschloo L Schoevers RA Borsboom D. Mental disorders as networks of problems: a review of recent insights. Soc Psychiatry Psychiatr Epidemiol. (2017) 52:1–10. doi: 10.1007/s00127-016-1319-z, PMID: 27921134 PMC5226976

[47] American Psychiatric Association. ed. Diagnostic and statistical manual of mental disorders: DSM-5. 5th ed. Washington, D.C.: American psychiatric association (2013). 947. p.

[48] Osman A Bagge CL Gutierrez PM Konick LC Kopper BA Barrios FX. The suicidal behaviors questionnaire-revised (SBQ-R):validation with clinical and nonclinical samples. Assessment. (2001) 8:443–54. doi: 10.1177/107319110100800409, PMID: 11785588

[49] Huen JMY Osman A Lew B Yip PSF. Utility of single items within the suicidal behaviors questionnaire-revised (SBQ-R): a bayesian network approach and relative importance analysis. Behav Sci. (2024) 14:410. doi: 10.3390/bs14050410, PMID: 38785901 PMC11117767

[50] Adjorlolo S Anum A Amin JM. Validation of the suicidal behaviors questionnaire-revised in adolescents in Ghana. J Ment Health. (2022) 31:302–8. doi: 10.1080/09638237.2020.1739239, PMID: 32174227

[51] Bernstein DP Fink L Handelsman L Foote J Lovejoy M Wenzel K. Initial reliability and validity of a new retrospective measure of child abuse and neglect. Am J Psychiatry. (1994) 151:1132–6. doi: 10.1176/ajp.151.8.1132, PMID: 8037246

[52] Fu w Yao s Yu h Zhao x Li ru Li y. Initial reliability and validity of childhood trauma questionnaire (CTQ-SF) applied in Chinese college students. Chin J Clin Psychol. (2005) 13(1):40–2. doi: 10.16128/j.cnki.1005-3611.2005.01.012

[53] Kroenke K Spitzer RL Williams JBW. The PHQ-9: validity of a brief depression severity measure. J Gen Intern Med. (2001) 16:606–13. doi: 10.1046/j.1525-1497.2001.016009606.x, PMID: 11556941 PMC1495268

[54] Richardson LP McCauley E Grossman DC McCarty CA Richards J Russo JE. Evaluation of the patient health questionnaire-9 item for detecting major depression among adolescents. Pediatrics. (2010) 126:1117–23. doi: 10.1542/peds.2010-0852, PMID: 21041282 PMC3217785

[55] Spitzer RL Kroenke K Williams JBW Löwe B. A brief measure for assessing generalized anxiety disorder: the GAD-7. Arch Intern Med. (2006) 166:1092. doi: 10.1001/archinte.166.10.1092, PMID: 16717171

[56] Sun J Liang K Chi X Chen S. Psychometric properties of the generalized anxiety disorder scale-7 item (GAD-7) in a large sample of chinese adolescents. Health Care (Don Mills). (2021) 9:1709. doi: 10.3390/healthcare9121709, PMID: 34946435 PMC8701121

[57] Epskamp S Fried EI. A tutorial on regularized partial correlation networks. Psychol Methods. (2018) 23:617–34. doi: 10.1037/met0000167, PMID: 29595293

[58] Epskamp S Cramer AOJ Waldorp LJ Schmittmann VD Borsboom D. qgraph: network visualizations of relationships in psychometric data. J Stat Softw. (2012) 48(4):1–18. doi: 10.18637/jss.v048.i04

[59] Opsahl T Agneessens F Skvoretz J. Node centrality in weighted networks: generalizing degree and shortest paths. Soc Networks. (2010) 32:245–51. doi: 10.1016/j.socnet.2010.03.006

[60] McNally RJ. Can network analysis transform psychopathology? Behav Res Ther. (2016) 86:95–104. doi: 10.1016/j.brat.2016.06.006, PMID: 27424882

[61] Jones PJ Ma R McNally RJ. Bridge centrality: a network approach to understanding comorbidity. Multivar Behav Res. (2021) 56:353–67. doi: 10.1080/00273171.2019.1614898, PMID: 31179765

[62] Garabiles MR Lao CK Xiong Y Hall BJ. Exploring comorbidity between anxiety and depression among migrant filipino domestic workers: a network approach. J Affect Disord. (2019) 250:85–93. doi: 10.1016/j.jad.2019.02.062, PMID: 30836284

[63] Li J Jin Y Xu S Luo X Wilson A Li H. Anxiety and depression symptoms among youth survivors of childhood sexual abuse: a network analysis. BMC Psychol. (2023) 11:278. doi: 10.1186/s40359-023-01275-3, PMID: 37717011 PMC10504753

[64] Luo J Bei D-L Zheng C Jin J Yao C Zhao J. The comorbid network characteristics of anxiety and depressive symptoms among chinese college freshmen. BMC Psychiatry. (2024) 24:297. doi: 10.1186/s12888-024-05733-z, PMID: 38641813 PMC11027377

[65] Fried EI Epskamp S Nesse RM Tuerlinckx F Borsboom D. What are “good” depression symptoms? Comparing the centrality of DSM and non-DSM symptoms of depression in a network analysis. J Affect Disord. (2016) 189:314–20. doi: 10.1016/j.jad.2015.09.005, PMID: 26458184

[66] Nyer M Mischoulon D Alpert JE Holt DJ Brill CD Yeung A. College students with depressive symptoms with and without fatigue: differences in functioning, suicidality, anxiety, and depressive severity. Ann Clin Psychiatry: Off J Am Acad Clin Psychiatr. (2015) 27:100–8., PMID: 25954936 PMC4539614

[67] Higson-Sweeney N Cooper K Dunn BD Loades ME. I’m always going to be tired”: a qualitative exploration of adolescents’ experiences of fatigue in depression. Eur Child Adolesc Psychiatry. (2024) 33:1369–81. doi: 10.1007/s00787-023-02243-3, PMID: 37300578 PMC10257178

[68] Uccella S Cordani R Salfi F Gorgoni M Scarpelli S Gemignani A. Sleep deprivation and insomnia in adolescence: implications for mental health. Brain Sci. (2023) 13:569. doi: 10.3390/brainsci13040569, PMID: 37190534 PMC10136689

[69] Colich NL McLaughlin KA. Accelerated pubertal development as a mechanism linking trauma exposure with depression and anxiety in adolescence. Curr Opin Psychol. (2022) 46:101338. doi: 10.1016/j.copsyc.2022.101338, PMID: 35430517 PMC9378424

[70] Stahl SM Zhang L Damatarca C Grady M. Brain circuits determine destiny in depression: a novel approach to the psychopharmacology of wakefulness, fatigue, and executive dysfunction in major depressive disorder. J Clin Psychiatry. (2003) 64 Suppl 14:6–17., PMID: 14658930

[71] Mills KL Goddings A-L Herting MM Meuwese R Blakemore S-J Crone EA. Structural brain development between childhood and adulthood: convergence across four longitudinal samples. Neuroimage. (2016) 141:273–81. doi: 10.1016/j.neuroimage.2016.07.044, PMID: 27453157 PMC5035135

[72] Beck AT Brown G Berchick RJ Stewart BL Steer RA. Relationship between hopelessness and ultimate suicide: a replication with psychiatric outpatients. Focus. (2006) 4:291–6. doi: 10.1176/foc.4.2.291, PMID: 2278535

[73] Breuer F Greggersen W Kahl KG Schweiger U Westermair AL. Caught in a web of trauma: network analysis of childhood adversity and adult mental ill-health. Child Abuse Negl. (2020) 107:104534. doi: 10.1016/j.chiabu.2020.104534, PMID: 32562964

[74] Egeland B Sroufe LA Erickson M. The developmental consequence of different patterns of maltreatment. Child Abuse Negl. (1983) 7:459–69. doi: 10.1016/0145-2134(83)90053-4, PMID: 6686797

[75] Yama MF Tovey SL Fogas BS. Childhood family environment and sexual abuse as predictors of anxiety and depression in adult women. Am J Orthopsychiatry. (1993) 63:136–41. doi: 10.1037/h0079399, PMID: 8427304

[76] Mack A. Childhood maltreatment predicts unfavorable course of illness and treatment outcome in depression: a meta-analysis. Yearb Psychiatry Appl Ment Health. (2013) 2013:32–3. doi: 10.1016/j.ypsy.2012.08.045, PMID: 22420036

[77] Beard C Millner AJ Forgeard MJC Fried EI Hsu KJ Treadway MT. Network analysis of depression and anxiety symptom relationships in a psychiatric sample. Psychol Med. (2016) 46:3359–69. doi: 10.1017/S0033291716002300, PMID: 27623748 PMC5430082

[78] Bai W Cai H Liu S Chen X Sha S Cheung T. Anxiety and depressive symptoms in college students during the late stage of the COVID-19 outbreak: a network approach. Transl Psychiatry. (2021) 11:638. doi: 10.1038/s41398-021-01738-4, PMID: 34921138 PMC8678580

[79] Jin Y Sha S Tian T Wang Q Liang S Wang Z. Network analysis of comorbid depression and anxiety and their associations with quality of life among clinicians in public hospitals during the late stage of the COVID-19 pandemic in China. J Affect Disord. (2022) 314:193–200. doi: 10.1016/j.jad.2022.06.051, PMID: 35780965 PMC9242942

[80] He L Wang J Zhang L Wang F Dong W Zhao W. Risk factors for anxiety and depressive symptoms in doctors during the coronavirus disease 2019 pandemic. Front Psychiatry. (2021) 12:687440. doi: 10.3389/fpsyt.2021.687440, PMID: 34220589 PMC8249942

[81] Brassard MR. Emotional and psychological abuse of children. Child Abuse Negl. (1994) 18:897–8. doi: 10.1016/0145-2134(94)90079-5

[82] Appleyard K Egeland B Van Dulmen MHM Alan L Sroufe. When more is not better: the role of cumulative risk in child behavior outcomes. J Child Psychol Psychiatry. (2005) 46:235–45. doi: 10.1111/j.1469-7610.2004.00351.x, PMID: 15755300

[83] Beck AT Weissman A Lester D Trexler L. The measurement of pessimism: the hopelessness scale. J Consult Clin Psychol. (1974) 42:861–5. doi: 10.1037/h0037562, PMID: 4436473

[84] Bagge CL Lamis DA Nadorff M Osman A. Relations between hopelessness, depressive symptoms and suicidality: mediation by reasons for living. J Clin Psychol. (2014) 70:18–31. doi: 10.1002/jclp.22005, PMID: 23798005

[85] Courtney E Kushwaha M Johnson J. Childhood emotional abuse and risk for hopelessness and depressive symptoms during adolescence. J Emot Abuse. (2008) 8:281–98. doi: 10.1080/10926790802262572

[86] Wang X Yang L Gao L Yang J Lei L Wang C. Childhood maltreatment and chinese adolescents’ bullying and defending: the mediating role of moral disengagement. Child Abuse Negl. (2017) 69:134–44. doi: 10.1016/j.chiabu.2017.04.016, PMID: 28460368

[87] Eckenrode J Campa MI Morris PA Henderson CR Bolger KE Kitzman H. The prevention of child maltreatment through the nurse family partnership program: mediating effects in a long-term follow-up study. Child Maltreat. (2017) 22:92–9. doi: 10.1177/1077559516685185, PMID: 28032513

[88] Yuan M He Y Wang F Wen X Tong Y Zhu D. Multi-level factors associated with psychological resilience in the face of adverse childhood experiences among chinese early adolescents. Child Abuse Negl. (2024) 153:106861. doi: 10.1016/j.chiabu.2024.106861, PMID: 38797118

[89] Hinojosa MS Hinojosa R. Positive and adverse childhood experiences and mental health outcomes of children. Child Abuse Negl. (2024) 149:106603. doi: 10.1016/j.chiabu.2023.106603, PMID: 38141478

[90] Qiao DP Chan YC. Child abuse in China: a yet-to-be-acknowledged “social problem” in the chinese mainland. Child Family Soc Work. (2005) 10:21–7. doi: 10.1111/j.1365-2206.2005.00347.x

[91] Jin J. Screening for depression and suicide risk in children and adolescents. JAMA. (2022) 328:1570. doi: 10.1001/jama.2022.18187, PMID: 36219439

[92] Sakurai H Uchida H Abe T Nakajima S Suzuki T Pollock BG. Trajectories of individual symptoms in remitters versus non-remitters with depression. J Affect Disord. (2013) 151:506–13. doi: 10.1016/j.jad.2013.06.035, PMID: 23886402

[93] De Arellano MAR Lyman DR Jobe-Shields L George P Dougherty RH Daniels AS. Trauma-focused cognitive-behavioral therapy for children and adolescents: assessing the evidence. Psychiatr Serv. (2014) 65:591–602. doi: 10.1176/appi.ps.201300255, PMID: 24638076 PMC4396183

[94] Li K Zhan X Ren L Liu N Zhang L Li L. The association of abuse and depression with suicidal ideation in chinese adolescents: a network analysis. Front Psychiatry. (2022) 13:853951. doi: 10.3389/fpsyt.2022.853951, PMID: 35418891 PMC8995894

