# Supplementary material for: Network analysis in depressed adolescents with suicidal ideation: the role of depression, anxiety, and childhood abuse
Source: Front Psychiatry. 2025 Aug 8;16:1645303. doi: 10.3389/fpsyt.2025.1645303 (PMC12370709; doi:10.3389/fpsyt.2025.1645303)
Supplement: Supplementary file 1 [file Supplementaryfile1.docx]

| Supplementary Table 1 The edge weights about this network | | | | | | | | | | | | | | | | | | | | | |
| --- | --- | --- | --- | --- | --- | --- | --- | --- | --- | --- | --- | --- | --- | --- | --- | --- | --- | --- | --- | --- | --- |
|  | PHQ1 | PHQ2 | PHQ3 | PHQ4 | PHQ5 | PHQ6 | PHQ7 | PHQ8 | PHQ9 | GAD1 | GAD2 | GAD3 | GAD4 | GAD5 | GAD6 | GAD7 | EA | PA | SA | EN | PN |
| PHQ1 | 0.00 |  |  |  |  |  |  |  |  |  |  |  |  |  |  |  |  |  |  |  |  |
| PHQ2 | 0.31 | 0.00 |  |  |  |  |  |  |  |  |  |  |  |  |  |  |  |  |  |  |  |
| PHQ3 | 0.00 | 0.01 | 0.00 |  |  |  |  |  |  |  |  |  |  |  |  |  |  |  |  |  |  |
| PHQ4 | 0.28 | 0.10 | 0.21 | 0.00 |  |  |  |  |  |  |  |  |  |  |  |  |  |  |  |  |  |
| PHQ5 | 0.05 | 0.08 | 0.16 | 0.25 | 0.00 |  |  |  |  |  |  |  |  |  |  |  |  |  |  |  |  |
| PHQ6 | 0.05 | 0.14 | 0.03 | 0.03 | 0.05 | 0.00 |  |  |  |  |  |  |  |  |  |  |  |  |  |  |  |
| PHQ7 | 0.00 | 0.00 | 0.05 | 0.12 | 0.05 | 0.03 | 0.00 |  |  |  |  |  |  |  |  |  |  |  |  |  |  |
| PHQ8 | 0.05 | 0.00 | 0.00 | 0.01 | 0.13 | 0.00 | 0.21 | 0.00 |  |  |  |  |  |  |  |  |  |  |  |  |  |
| PHQ9 | 0.00 | 0.16 | 0.05 | 0.00 | 0.02 | 0.23 | 0.05 | 0.03 | 0.00 |  |  |  |  |  |  |  |  |  |  |  |  |
| GAD1 | 0.00 | 0.11 | 0.01 | 0.06 | 0.01 | 0.00 | 0.00 | 0.00 | 0.04 | 0.00 |  |  |  |  |  |  |  |  |  |  |  |
| GAD2 | 0.01 | 0.03 | 0.02 | 0.02 | 0.02 | 0.01 | 0.00 | 0.04 | 0.05 | 0.36 | 0.00 |  |  |  |  |  |  |  |  |  |  |
| GAD3 | 0.00 | 0.00 | 0.00 | 0.03 | 0.00 | 0.08 | 0.03 | 0.00 | 0.00 | 0.00 | 0.31 | 0.00 |  |  |  |  |  |  |  |  |  |
| GAD4 | 0.06 | 0.03 | 0.00 | 0.02 | 0.00 | 0.00 | 0.02 | 0.00 | 0.03 | 0.15 | 0.17 | 0.19 | 0.00 |  |  |  |  |  |  |  |  |
| GAD5 | 0.00 | 0.00 | 0.05 | 0.00 | 0.01 | 0.00 | 0.07 | 0.23 | 0.02 | 0.12 | 0.00 | 0.02 | 0.18 | 0.00 |  |  |  |  |  |  |  |
| GAD6 | 0.03 | 0.03 | 0.04 | 0.03 | 0.00 | 0.09 | 0.00 | 0.09 | 0.00 | 0.16 | 0.08 | 0.05 | 0.11 | 0.14 | 0.00 |  |  |  |  |  |  |
| GAD7 | 0.00 | 0.00 | 0.00 | 0.00 | 0.00 | 0.07 | 0.03 | 0.00 | 0.07 | 0.04 | 0.05 | 0.18 | 0.00 | 0.15 | 0.03 | 0.00 |  |  |  |  |  |
| EA | 0.00 | 0.01 | 0.00 | 0.00 | 0.00 | 0.04 | 0.04 | 0.07 | 0.13 | 0.00 | 0.00 | 0.00 | 0.00 | 0.00 | 0.00 | 0.02 | 0.00 |  |  |  |  |
| PA | 0.00 | 0.00 | 0.00 | 0.00 | 0.00 | 0.00 | 0.00 | 0.00 | 0.00 | 0.00 | 0.00 | 0.00 | 0.00 | 0.00 | 0.00 | 0.00 | 0.32 | 0.00 |  |  |  |
| SA | 0.00 | 0.00 | 0.00 | 0.00 | 0.00 | 0.00 | 0.00 | 0.00 | 0.00 | 0.00 | 0.00 | 0.00 | 0.00 | 0.00 | 0.00 | 0.00 | 0.09 | 0.09 | 0.00 |  |  |
| EN | 0.00 | 0.00 | 0.01 | 0.00 | 0.01 | 0.00 | 0.00 | 0.00 | 0.04 | 0.00 | 0.00 | 0.00 | 0.00 | 0.00 | 0.00 | 0.00 | 0.31 | 0.05 | 0.00 | 0.00 |  |
| PN | 0.00 | 0.00 | 0.00 | 0.00 | 0.00 | 0.00 | 0.00 | 0.01 | 0.00 | 0.00 | 0.00 | 0.00 | 0.00 | 0.00 | 0.00 | 0.03 | 0.04 | 0.09 | 0.04 | 0.48 | 0.00 |
