# Supplementary material for: Network analysis in depressed adolescents with suicidal ideation: the role of depression, anxiety, and childhood abuse
Source: Front Psychiatry. 2025 Aug 8;16:1645303. doi: 10.3389/fpsyt.2025.1645303 (PMC12370709; doi:10.3389/fpsyt.2025.1645303)
Supplement: Supplementary file 2 [file Supplementaryfile2.docx]

Supplementary Material

# Supplementary Figures and Tables


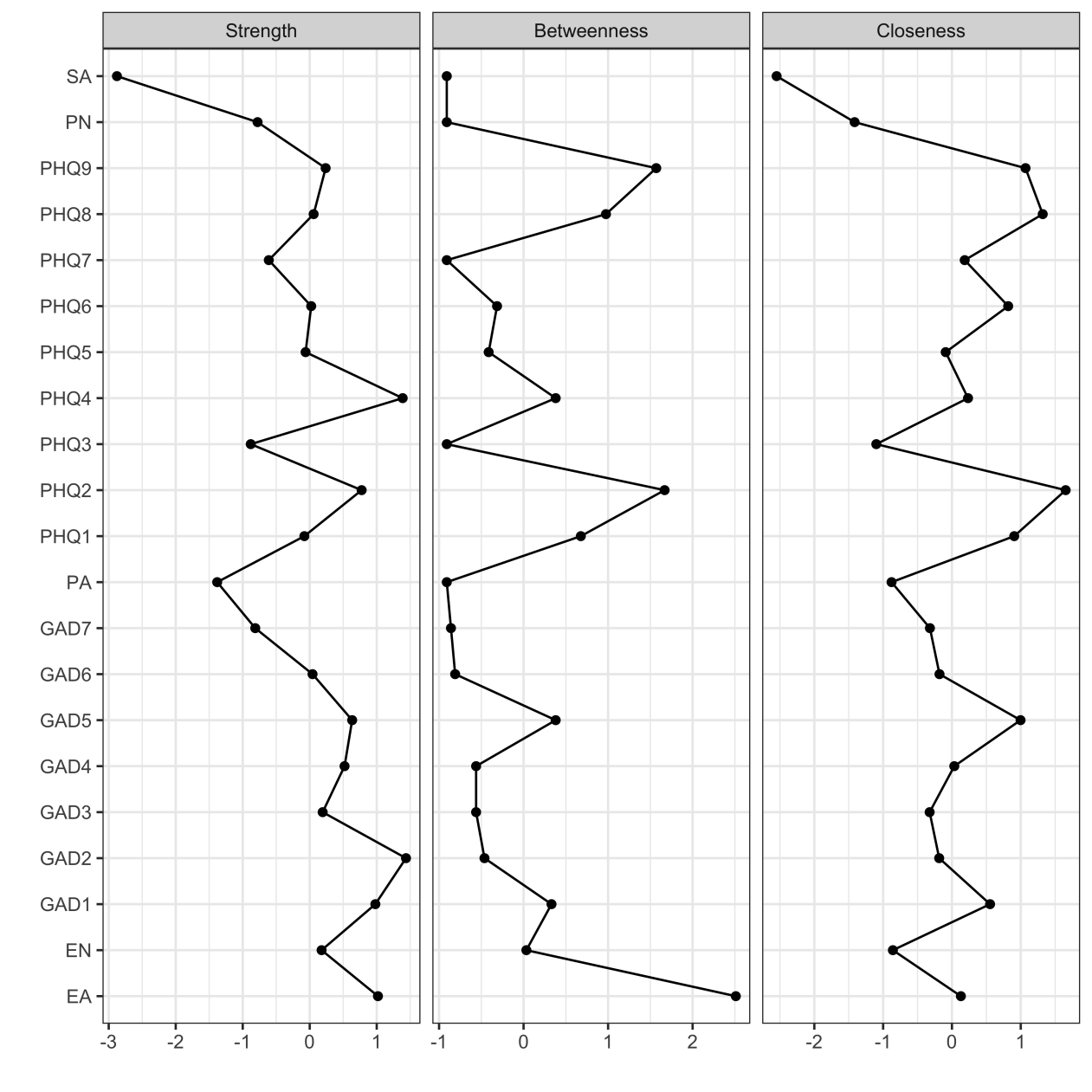


**Supplementary Figure 1.** Centrality plots for the 21 nodes depicted as strength, betweenness, and closeness. Strength refers to the sum of the absolute values of the weights of the edges connected to a node. Closeness measures the average distance from a node to all other nodes in the network. It indicates how quickly information can spread from a given node to the rest of the network. Betweenness quantifies the extent to which a node lies on the shortest path between other nodes, reflecting its role as a bridge or mediator within the network.


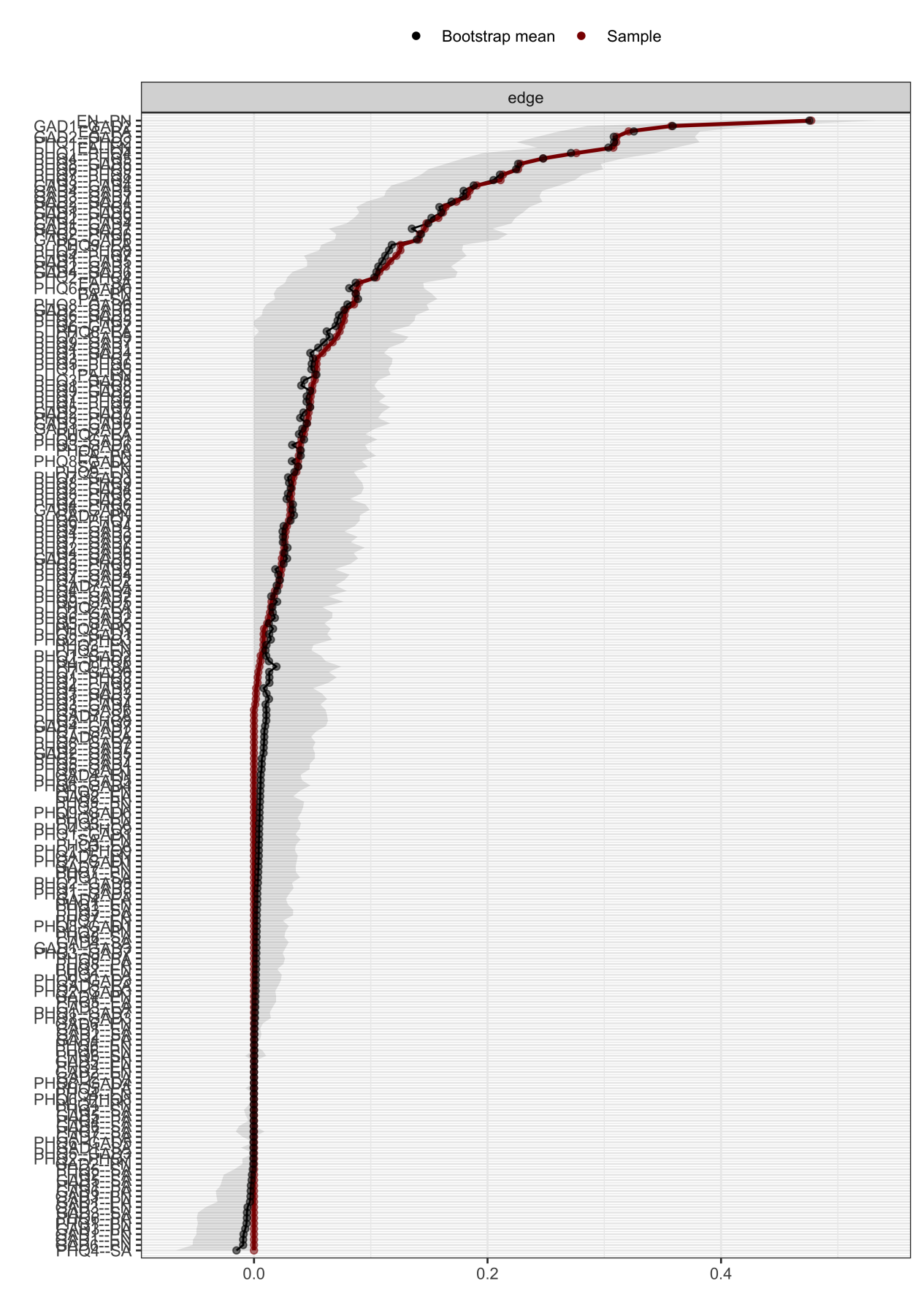


**Supplementary Figure 2.** Bootstrapped confidence intervals (CIs) of the edge weights in the network. The CI width reflects estimate precision, with greater overlap between intervals indicating higher network model accuracy. Narrower CIs imply more reliable network structure.


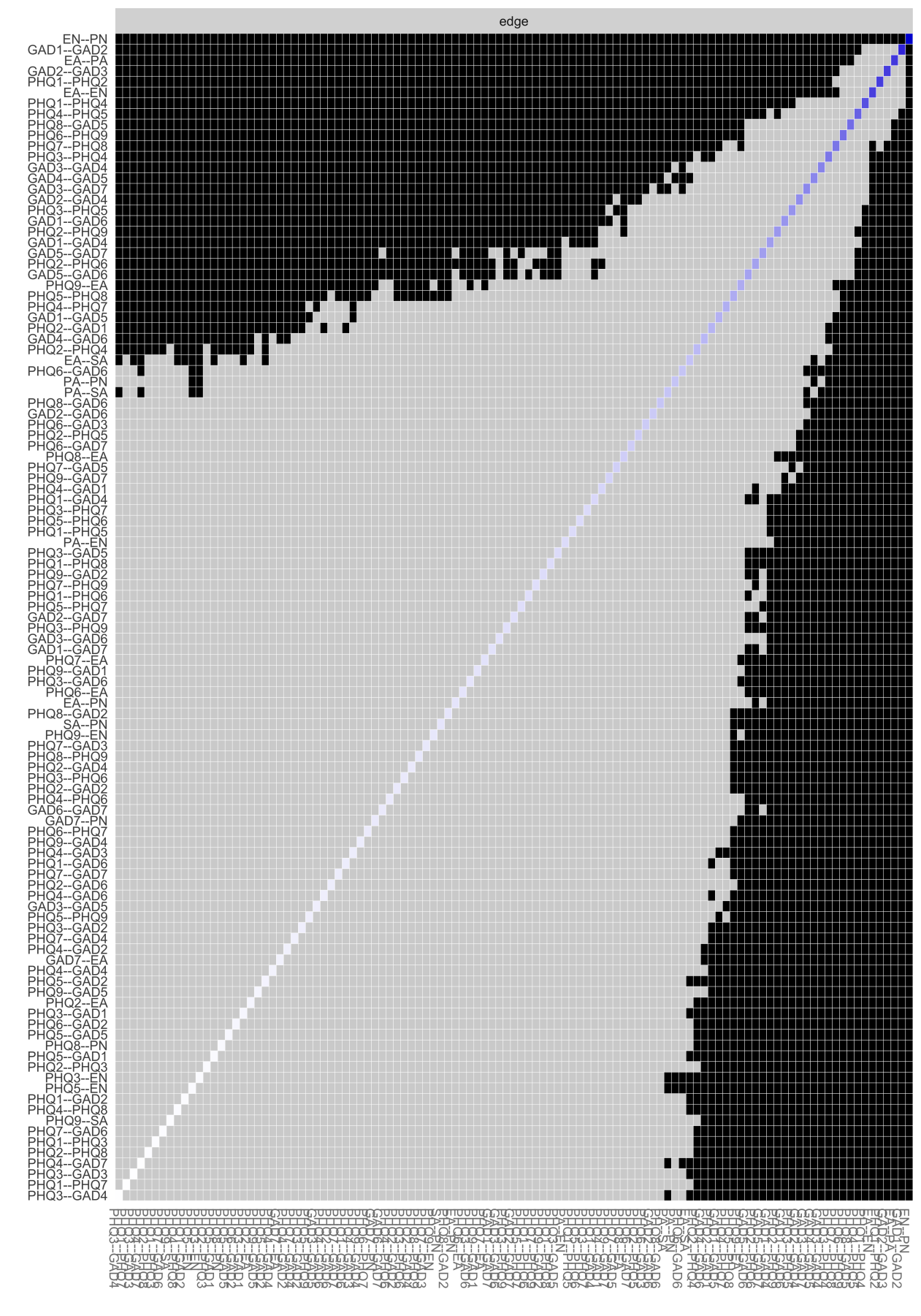


**Supplementary Figure 3.** Confidence intervals around edges between Depression, Anxiety, and Childhood Abuse symptoms.


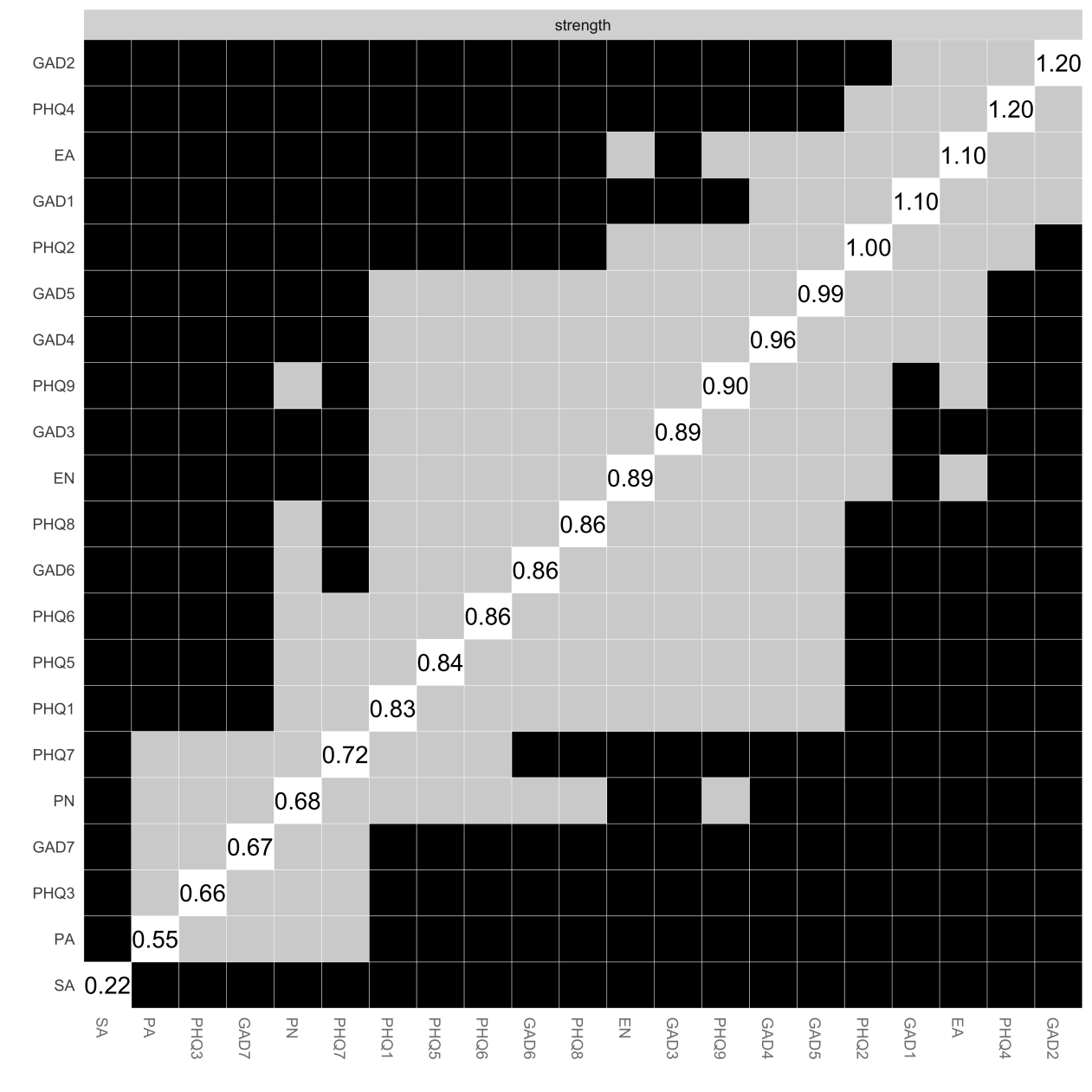


**Supplementary Figure 4.** Bootstrapped difference tests results between node strength.It shows strength of key nodes is significantly greater than that of other nodes.

**Supplementary Figure 5.**
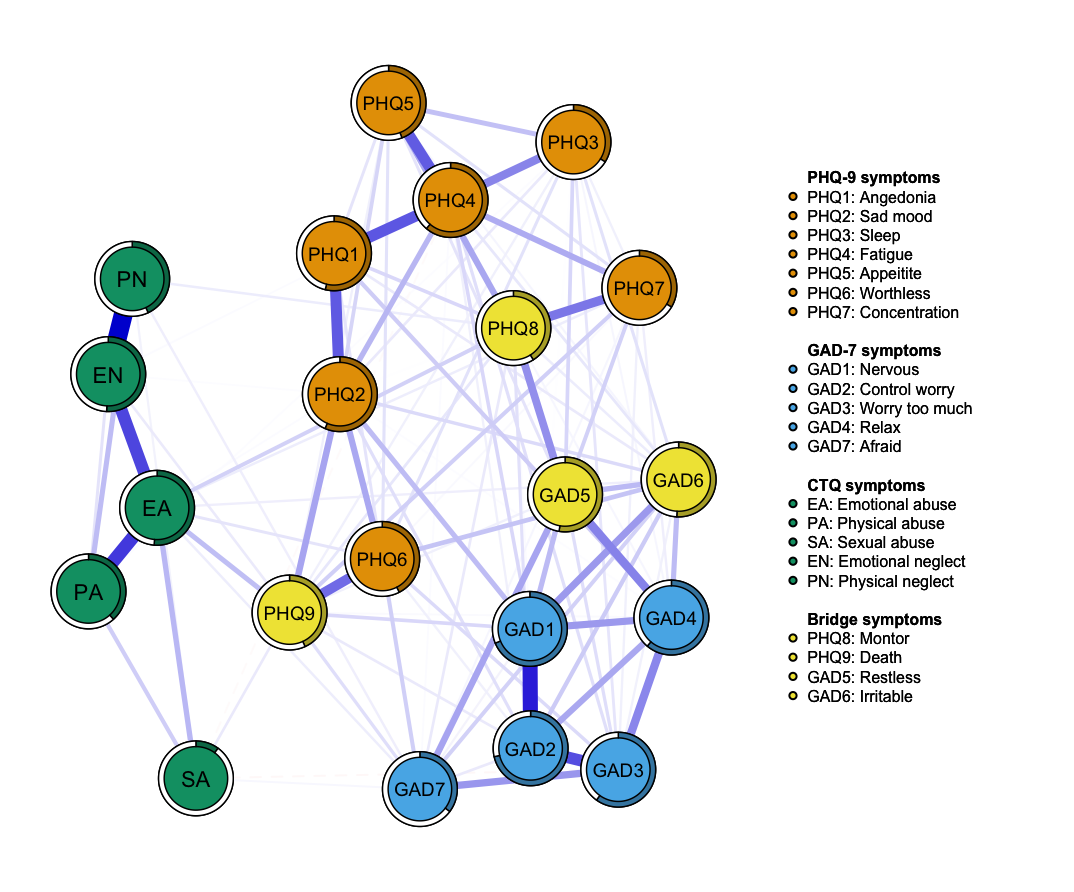
 **The network displaying the relationship between depression, anxiety, and childhood abuse symptoms of the Han ethnicity samples.**


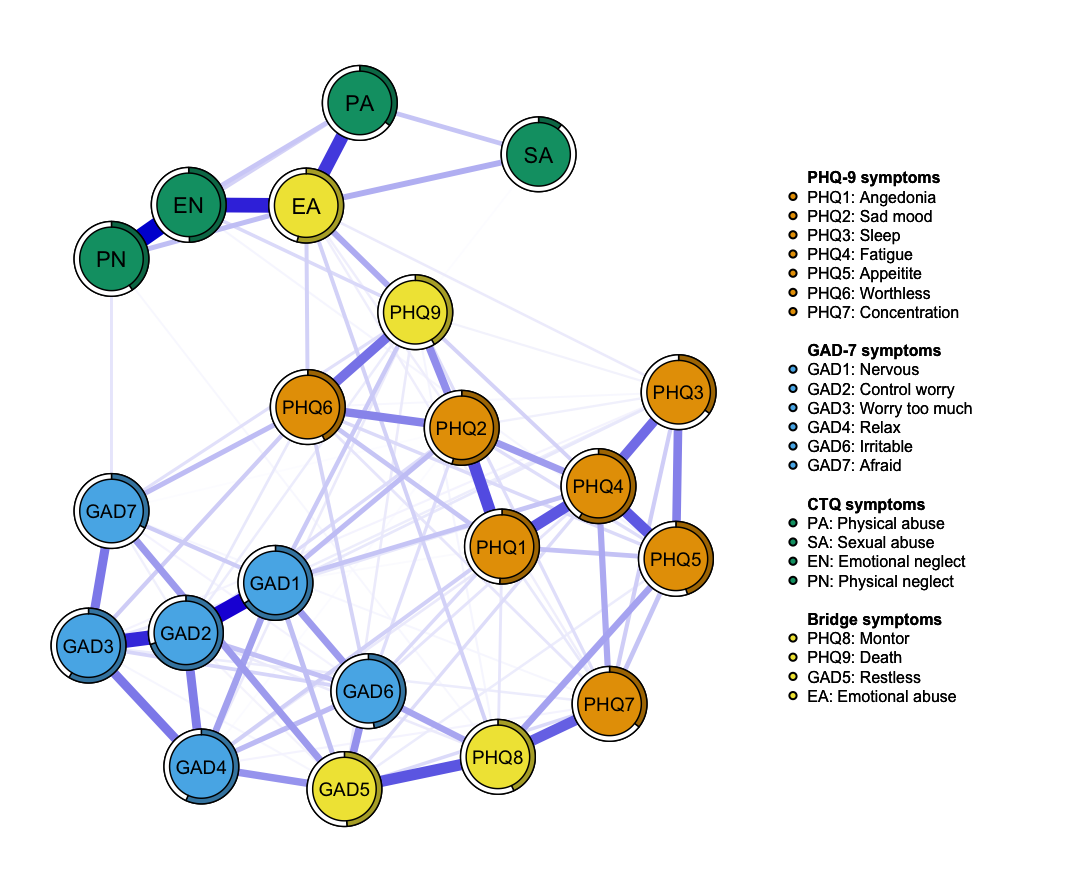
**Supplementary Figure 6. The network displaying the relationship between depression, anxiety, and childhood abuse symptoms of the urban samples.**


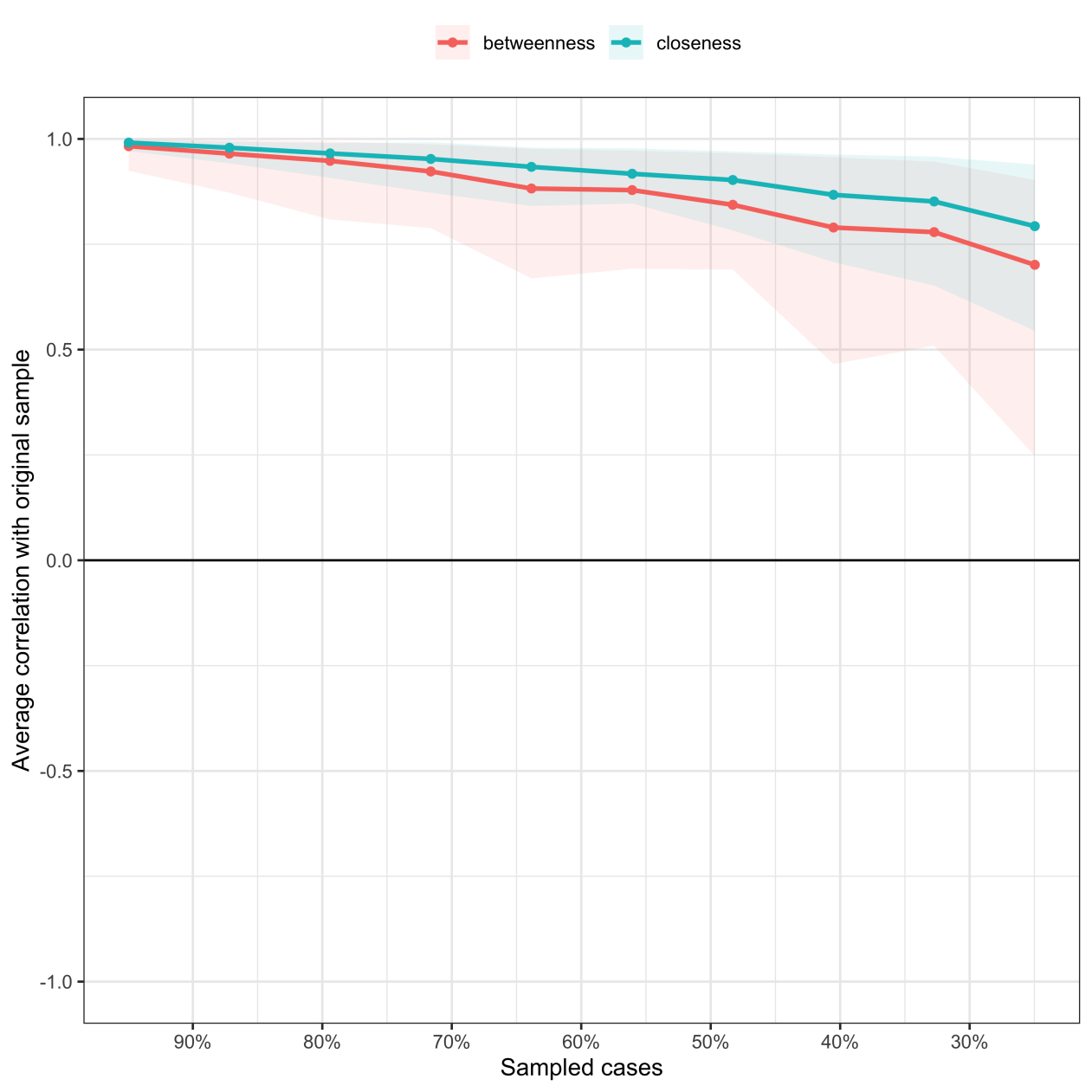


**Supplementary Figure 7.** The stability of node betweenness and closeness indices using case-dropping bootstrap. According to established guidelines, a CS coefficient value greater than 0.25 is considered acceptable, indicating that the centrality measures are reasonably stable. A CS coefficient value greater than 0.5 is considered excellent, signifying a high degree of stability and reliability in the centrality measures.


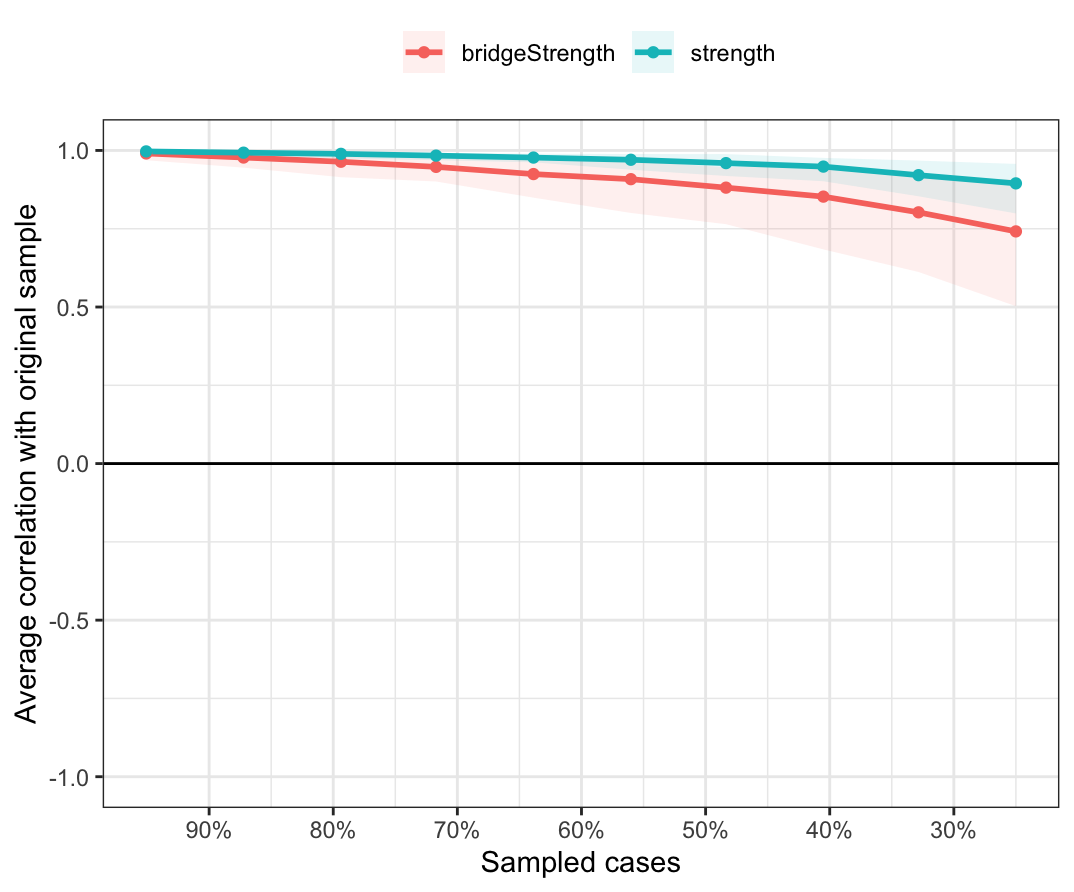


**Supplementary Figure 8. The stability of centrality and bridge centrality indices using case-dropping bootstrap of the Han ethnicity samples.**

**
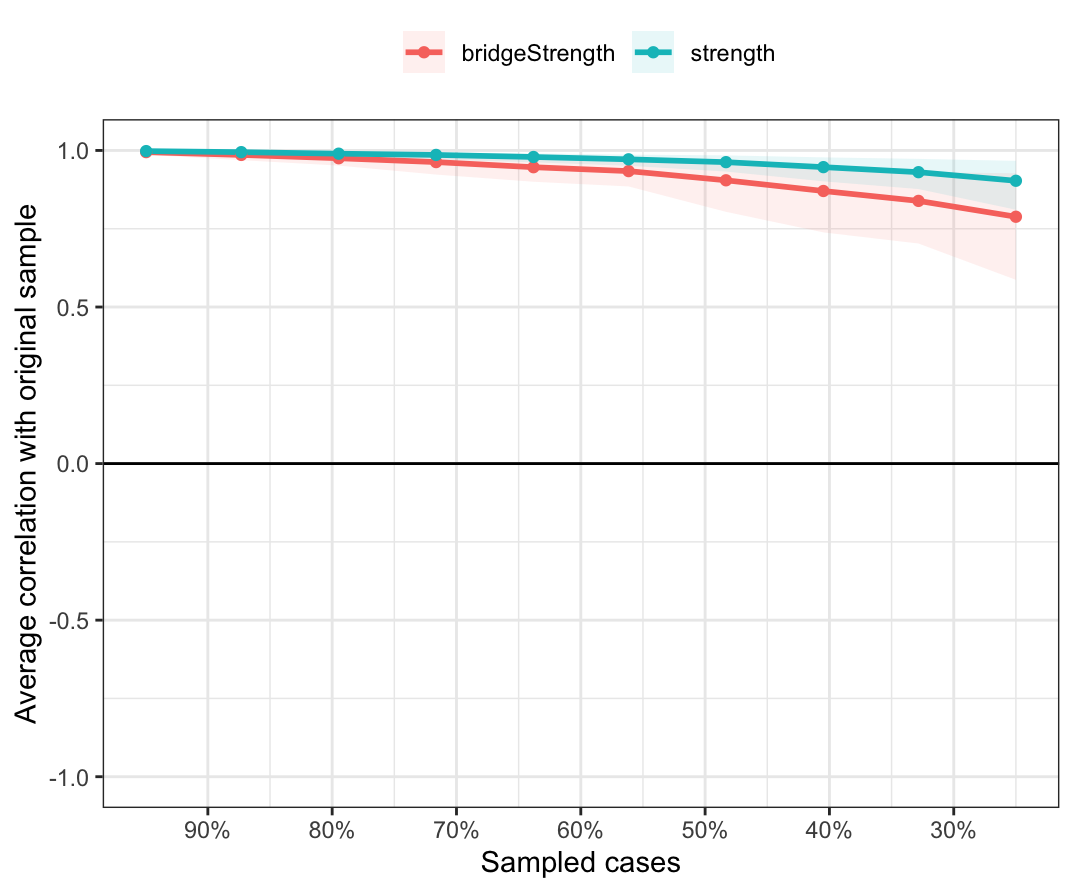
**

**Supplementary Figure 9. The stability of centrality and bridge centrality indices using case-dropping bootstrap of the urban samples.**

| Supplementary Table 1 The edge weights about this network | | | | | | | | | | | | | | | | | | | | | |
| --- | --- | --- | --- | --- | --- | --- | --- | --- | --- | --- | --- | --- | --- | --- | --- | --- | --- | --- | --- | --- | --- |
|  | PHQ1 | PHQ2 | PHQ3 | PHQ4 | PHQ5 | PHQ6 | PHQ7 | PHQ8 | PHQ9 | GAD1 | GAD2 | GAD3 | GAD4 | GAD5 | GAD6 | GAD7 | EA | PA | SA | EN | PN |
| PHQ1 | 0.00 |  |  |  |  |  |  |  |  |  |  |  |  |  |  |  |  |  |  |  |  |
| PHQ2 | 0.31 | 0.00 |  |  |  |  |  |  |  |  |  |  |  |  |  |  |  |  |  |  |  |
| PHQ3 | 0.00 | 0.01 | 0.00 |  |  |  |  |  |  |  |  |  |  |  |  |  |  |  |  |  |  |
| PHQ4 | 0.28 | 0.10 | 0.21 | 0.00 |  |  |  |  |  |  |  |  |  |  |  |  |  |  |  |  |  |
| PHQ5 | 0.05 | 0.08 | 0.16 | 0.25 | 0.00 |  |  |  |  |  |  |  |  |  |  |  |  |  |  |  |  |
| PHQ6 | 0.05 | 0.14 | 0.03 | 0.03 | 0.05 | 0.00 |  |  |  |  |  |  |  |  |  |  |  |  |  |  |  |
| PHQ7 | 0.00 | 0.00 | 0.05 | 0.12 | 0.05 | 0.03 | 0.00 |  |  |  |  |  |  |  |  |  |  |  |  |  |  |
| PHQ8 | 0.05 | 0.00 | 0.00 | 0.01 | 0.13 | 0.00 | 0.21 | 0.00 |  |  |  |  |  |  |  |  |  |  |  |  |  |
| PHQ9 | 0.00 | 0.16 | 0.05 | 0.00 | 0.02 | 0.23 | 0.05 | 0.03 | 0.00 |  |  |  |  |  |  |  |  |  |  |  |  |
| GAD1 | 0.00 | 0.11 | 0.01 | 0.06 | 0.01 | 0.00 | 0.00 | 0.00 | 0.04 | 0.00 |  |  |  |  |  |  |  |  |  |  |  |
| GAD2 | 0.01 | 0.03 | 0.02 | 0.02 | 0.02 | 0.01 | 0.00 | 0.04 | 0.05 | 0.36 | 0.00 |  |  |  |  |  |  |  |  |  |  |
| GAD3 | 0.00 | 0.00 | 0.00 | 0.03 | 0.00 | 0.08 | 0.03 | 0.00 | 0.00 | 0.00 | 0.31 | 0.00 |  |  |  |  |  |  |  |  |  |
| GAD4 | 0.06 | 0.03 | 0.00 | 0.02 | 0.00 | 0.00 | 0.02 | 0.00 | 0.03 | 0.15 | 0.17 | 0.19 | 0.00 |  |  |  |  |  |  |  |  |
| GAD5 | 0.00 | 0.00 | 0.05 | 0.00 | 0.01 | 0.00 | 0.07 | 0.23 | 0.02 | 0.12 | 0.00 | 0.02 | 0.18 | 0.00 |  |  |  |  |  |  |  |
| GAD6 | 0.03 | 0.03 | 0.04 | 0.03 | 0.00 | 0.09 | 0.00 | 0.09 | 0.00 | 0.16 | 0.08 | 0.05 | 0.11 | 0.14 | 0.00 |  |  |  |  |  |  |
| GAD7 | 0.00 | 0.00 | 0.00 | 0.00 | 0.00 | 0.07 | 0.03 | 0.00 | 0.07 | 0.04 | 0.05 | 0.18 | 0.00 | 0.15 | 0.03 | 0.00 |  |  |  |  |  |
| EA | 0.00 | 0.01 | 0.00 | 0.00 | 0.00 | 0.04 | 0.04 | 0.07 | 0.13 | 0.00 | 0.00 | 0.00 | 0.00 | 0.00 | 0.00 | 0.02 | 0.00 |  |  |  |  |
| PA | 0.00 | 0.00 | 0.00 | 0.00 | 0.00 | 0.00 | 0.00 | 0.00 | 0.00 | 0.00 | 0.00 | 0.00 | 0.00 | 0.00 | 0.00 | 0.00 | 0.32 | 0.00 |  |  |  |
| SA | 0.00 | 0.00 | 0.00 | 0.00 | 0.00 | 0.00 | 0.00 | 0.00 | 0.00 | 0.00 | 0.00 | 0.00 | 0.00 | 0.00 | 0.00 | 0.00 | 0.09 | 0.09 | 0.00 |  |  |
| EN | 0.00 | 0.00 | 0.01 | 0.00 | 0.01 | 0.00 | 0.00 | 0.00 | 0.04 | 0.00 | 0.00 | 0.00 | 0.00 | 0.00 | 0.00 | 0.00 | 0.31 | 0.05 | 0.00 | 0.00 |  |
| PN | 0.00 | 0.00 | 0.00 | 0.00 | 0.00 | 0.00 | 0.00 | 0.01 | 0.00 | 0.00 | 0.00 | 0.00 | 0.00 | 0.00 | 0.00 | 0.03 | 0.04 | 0.09 | 0.04 | 0.48 | 0.00 |
